# Neuropilin 1 is an entry factor that promotes EBV infection of nasopharyngeal epithelial cells

**DOI:** 10.1038/ncomms7240

**Published:** 2015-02-11

**Authors:** Hong-Bo Wang, Hua Zhang, Jing-Ping Zhang, Yan Li, Bo Zhao, Guo-Kai Feng, Yong Du, Dan Xiong, Qian Zhong, Wan-Li Liu, Huamao Du, Man-Zhi Li, Wen-Lin Huang, Sai Wah Tsao, Lindsey Hutt-Fletcher, Yi-Xin Zeng, Elliott Kieff, Mu-Sheng Zeng

**Affiliations:** 1 State Key Laboratory of Oncology in South China, Collaborative Innovation Center for Cancer Medicine, Department of Experimental Research, Sun Yat-sen University Cancer Center, Guangzhou, Guangdong 510060, People’s Republic of China; 2 Department of Medicine and Microbiology and Molecular Genetics, Channing Laboratory, Brigham and Women’s Hospital and Harvard Medical School, Boston, Massachusetts 02115, USA; 3 College of Biotechnology, Southwest University, Chongqing 400715, People’s Republic of China; 4 Department of Anatomy and Center for Cancer Research, University of Hong Kong, Hong Kong, SAR, People’s Republic of China; 5 Department of Microbiology and Immunology, Louisiana State University, Health Science Center, Shreveport, Louisiana 71130, USA

## Abstract

Epstein–Barr virus (EBV) is implicated as an aetiological factor in B lymphomas and nasopharyngeal carcinoma. The mechanisms of cell-free EBV infection of nasopharyngeal epithelial cells remain elusive. EBV glycoprotein B (gB) is the critical fusion protein for infection of both B and epithelial cells, and determines EBV susceptibility of non-B cells. Here we show that neuropilin 1 (NRP1) directly interacts with EBV gB^23–431^. Either knockdown of NRP1 or pretreatment of EBV with soluble NRP1 suppresses EBV infection. Upregulation of NRP1 by overexpression or EGF treatment enhances EBV infection. However, NRP2, the homologue of NRP1, impairs EBV infection. EBV enters nasopharyngeal epithelial cells through NRP1-facilitated internalization and fusion, and through macropinocytosis and lipid raft-dependent endocytosis. NRP1 partially mediates EBV-activated EGFR/RAS/ERK signalling, and NRP1-dependent receptor tyrosine kinase (RTK) signalling promotes EBV infection. Taken together, NRP1 is identified as an EBV entry factor that cooperatively activates RTK signalling, which subsequently promotes EBV infection in nasopharyngeal epithelial cells.

## Abstract (web summary)

Epstein–Barr virus (EBV) is involved in the development of some cancers including nasopharyngeal carcinoma. Here, the authors show that a direct interaction between the viral protein gB and a host protein, neuropilin 1, is required for EBV infection of nasopharyngeal epithelial cells.

## Graphical abstract

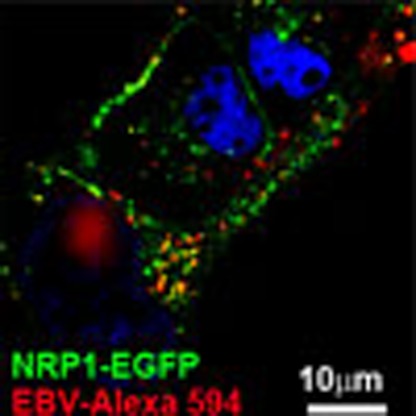

Epstein–Barr virus (EBV) is a ubiquitous human herpesvirus 4 (HHV4) that establishes latent infections in >90% of the adult population worldwide^1^,^2^. EBV is implicated as an aetiological factor in multiple malignancies of either lymphoid or epithelial origin, including Burkitt lymphoma, Hodgkin’s lymphoma, gastric carcinoma and nasopharyngeal carcinoma (NPC), suggesting its primary tropism for these cells^2^,^3^. The mechanism involved in EBV infection of B cells has been well elucidated, while the mechanisms of EBV infection of epithelial cells remain elusive, mainly due to the lack of representative cell model that are highly susceptible to cell-free EBV infection^4^,^5^,^6^.

EBV infection of B cells consists of at least two distinct mechanistic steps^7^. EBV attaches to the targeted cells through the interaction of EBV glycoprotein gp350/220 with CD21 (the B cell complement receptor, CR2) or CD35 (refs 8, 9). Subsequently, EBV fuses and penetrates into B cells, triggered by the interaction of gp42 (an additional EBV glycoprotein) with HLA class II, in the presence of EBV gB and gHgL (the core fusion machinery)^10^. However, the binding receptors CD21 and CD35, and the fusion receptor HLA class II, are expressed at low or undetectable levels in epithelial cells^11^,^12^. Therefore, EBV gp42 and gp350 were not essential in EBV infection of epithelial cells, suggesting different mechanisms contributing to EBV infection of epithelial cells^12^.

EBV gB is the most highly conserved glycoprotein required for membrane fusion in herpesviruses, but its cellular mediator involved in EBV fusion has not been identified so far^13^. EBV strains with higher expression of gB exhibit an increased capacity to infect cells that are normally refractory to EBV infection^14^. EBV gB contains a consensus furin cleavage site^15^,^16^. After cleavage by furin, EBV gB exhibited as a N-terminal peptide with 78 kDa, and a C-terminal peptide with 58 kDa. Both full-length and furin-cleaved gB are moderately abundant potential fusogens in mature EBV envelopes^16^. Deletion of the consensus furin cleavage site of gB, which is speculated to be a potential cryptic CendR motif, results in the suppression of cell-cell fusion, indicating the importance of this site to EBV infection^15^. Peptides that expose the CendR motif with the consensus sequence R/K/XXR/K at the C-terminus bind to Neuropilin 1 (NRP1) and are internalized into the cell^17^,^18^.

NRP1, as a co-receptor for class III semaphorins and multiple growth factors, such as EGF, VEGF, PDGF, HGF, TGF-β and FGF, cooperatively enhances the activity of the receptor tyrosine kinases (RTKs)^19^. In addition, NRP1 mediates the penetration of iRGD conjugated nanoparticles into tissue and cells through functioning as a receptor for CendR motif, the proteolytic cleavage products of iRGD after binding to integrins^17^,^20^. Multiple viruses possess CendR motifs within their capsid proteins and may undergo proteolytic cleavage to expose the CendR motif to be infective^18^. Human T-cell lymphotropic virus type 1 (HTLV-1) is one of such virus that bind to and internalize into immune cells via the interaction with NRP1 and its surface subunit (SU) containing a CendR motif (KPXR)^21^,^22^.

Together, these observations led us to deduce that NRP1 might serve as an unidentified entry factor or a cellular mediator for gB during EBV infection. Here, we demonstrate that NRP1 interacts with EBV gB and promotes EBV infection of epithelial cells by coordinating the RTK signalling pathway and macropinocytic events.

## Results

### EBV gB directly interacts with NRP1

**Figure 1:**
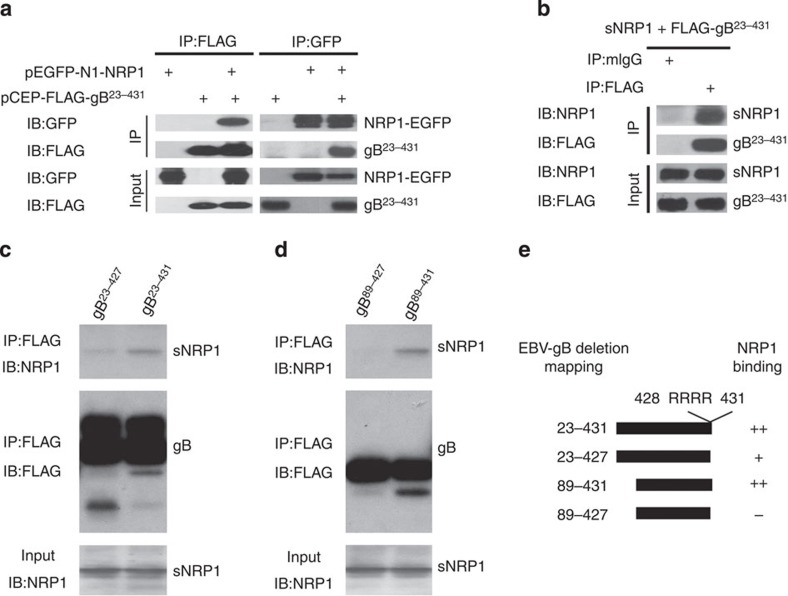
NRP1 mediates EBV infection through interaction with EBV-gB. (**a**) NRP1 co-immunoprecipitated with EBV gB. HEK-293FT cells co-transfected with pEGFP-NRP1 (EGFP-tagged NRP1) and pCEP-FLAG-gB^23–431^ (FLAG-labelled gB^23–431^) for 36 h were immunoprecipitated (IP) with an antibody against FLAG or GFP, followed by immunoblotting (IB) analysis for the complex with the indicated antibody. (**b**) Purified EBV-gB^23–431^ interacted with soluble NRP1 (sNRP1) *in vitro*. FLAG-gB^23–431^, purified from the supernatant of HEK-293FT-pCEP-FLAG-gB^23–431^ stable cell line, was incubated with the commercial sNRP1. The protein complex was captured with the anti-FLAG or mIgG (control) and analysed by immunoblotting with anti-NRP1 or anti-FLAG antibodies. Input is the purified EBV-gB^23–431^ and sNRP1. (**c**,**d**) gB^428–431^ (CendR motif) is an important element for gB to interact with NRP1 produced by an *in vitro* transcription–translation system. The cell lysates from HEK-293FT cells transiently transfected with pCEP-FLAG-gB^23–427^, pCEP-FLAG-gB^23–431^, pCEP-FLAG-gB^89–427^ or pCEP-FLAG-gB^89–431^ were immunoprecipitated with the anti-FLAG (M2) Sepharose beads, as indicated. The M2-precipitated products were incubated with the soluble NRP1^30–636^ (sNRP1^30–636^) expressed by an *in vitro* transcription–translation system. The immune complex was analysed by immunoblotting (IB) with anti-NRP1 or anti-FLAG antibodies. (**e**) Schematic summary of NRP1 and gB interactions.

Multiple viruses, including EBV, possess CendR motifs^18^, a structure that specifically mediates NRP1—dependent tissue and cell penetration. To examine the potential physical interaction of gB with NRP1, co-immunoprecipitations were performed in HEK-293FT cells transfected with expression plasmids for both NRP1 (NRP1-EGFP) and the CendR motif exposed gB^23–431^ (FLAG-gB). Consistent with the previous reports about the crystal structure analysis^23^, gB^23–431^ mainly presented as the trimeric form, determined by either western blotting analysis of the natural form of gB^23–431^ in DSS cross-linked gB-overexpressing cells or native–PAGE analysis of the purified pulled-down gB (Supplementary Fig. 1). Immuoprecipitation with either anti-FLAG or anti-EGFP antibody demonstrated the physical interaction of gB^23–431^ and NRP1 (Fig. 1a). Furthermore, this interaction acted in a direct manner, confirmed by an *in vitro* binding assay of the commercial soluble NRP1 (sNRP1) and FLAG-gB^23–431^ isolated from the supernatants of HEK-293FT cells (Fig. 1b).

To investigate whether the CendR motif (gB^428-431^) mediates the interaction between NRP1 and gB, the interaction between NRP1 and gB mutants with various deletion was determined by an *in vitro* binding assay. The CendR motif—deletion mutant gB^23–427^ still retained binding affinity to NRP1, although the binding was substantially reduced in comparison to that of the CendR motif—containing gB^23–431^ (Fig. 1c), indicating that in addition to the CendR motif another element may also contribute to the interaction of gB and NRP1. Reimer *et al*.^24^ reported that the insertion of a 5-amino-acid sequence at residue 88 of gB abolished cell-cell fusion activity. Therefore, gB^89–431^ and gB^89–427^, the mutants with deletion of gB^23-88^, were further analysed for their interaction with NRP1. EBV gB^89–427^, with deletion of both gB^23–88^ and gB^428–431^ (the CendR motif), almost has no interaction with NRP1 (Fig. 1d). These data suggested that both gB^23–88^ and gB^428–431^ (the CendR motif) might be involved in the interaction between NRP1 and gB.

### NRP1 enhances EBV entry into nasopharyngeal epithelial cells

Given the physical interaction of NRP1 and gB, we proceeded to determine whether NRP1 mediated EBV infection of nasopharyngeal epithelial cells. EBV is present in the most undifferentiated NPC tumor cells and is occasionally detected in the pre-invasive lesions^2^,^25^,^26^,^27^. However, nasopharyngeal epithelial cells are relatively refractory to cell-free EBV infection^5^,^28^. We first screened primary nasopharyngeal epithelial cells (NPEC03 and NPECw), immortalized nasopharyngeal epithelial cells (NP69, NPEC1-Bmi1 and NPEC2-Bmi1) and nasopharyngeal cancer cells (SUNE1, SUNE2, 6-10B, CNE1, CNE2 and HNE1) to identify cell lines with higher infection efficiency by using the recombinant Akata GFP-EBV, which expresses high levels of gB. The full-length and cleaved forms of gB were found to be present in the purified virus (Supplementary Fig. 2). The primary cells (NPEC03), immortalized cells (NPEC1-Bmi1 and NPEC2-Bmi1) and NPC cancer cells (HNE1) were relatively sensitive to cell-free EBV infection, with the infection rate ranging from 10 to 20%. (Supplementary Fig. 3a). Hence, NPEC1-Bmi1 and HNE1 cells were used for most of the further infection experiments. The expressions of CD21 and CD35, which are essential for EBV infection of B cells^8^,^9^, were undetectable in these cells, determined by real-time PCR (Supplementary Fig. 3b,c). In addition, EBV infection could not be inhibited by the blocking antibody against gp350 (Supplementary Fig. 4). These data suggested that EBV infection of nasopharyngeal epithelial cells was not dependent on CD21, CD35 and gp350.

**Figure 2:**
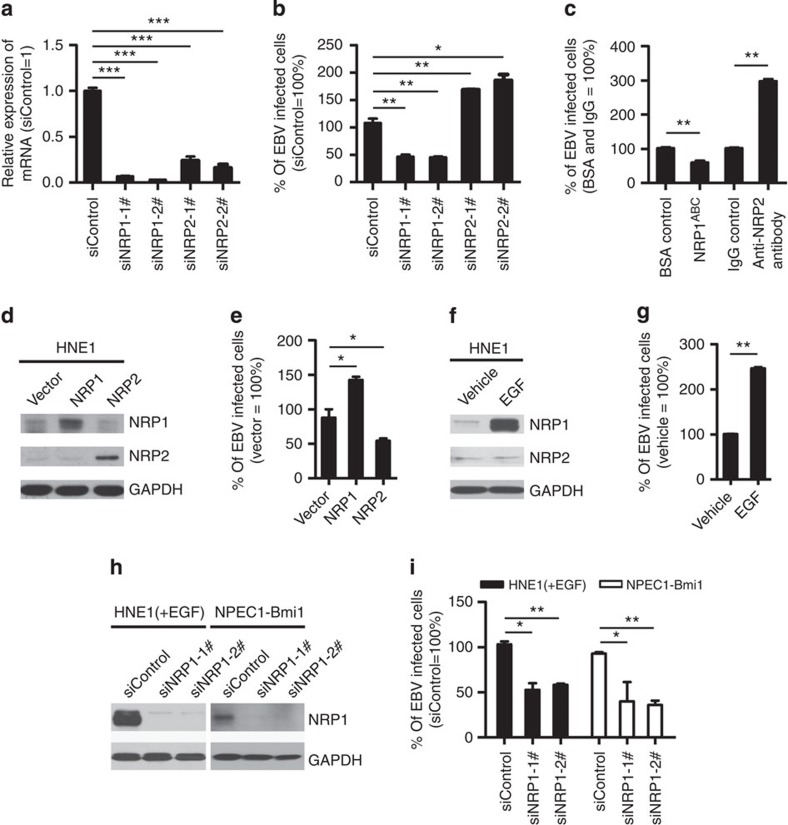
NRP1 enhances EBV infection, while NRP2 suppresses EBV infection. (**a**,**b**) Downregulation of NRP1 impaired, whereas knockdown of NRP2 promoted EBV infection. HNE1 cells were transfected with siRNA duplexes targeting NRP1 or NRP2 for 48 h, followed by NRP expression analysis by real-time PCR (**a**) or analysis for the efficiency of EBV infection (**b**); *n*=3. (**c**) EBV infection was blocked by soluble NRP1^ABC^, but enhanced by antibody against NRP2. For NRP1^ABC^ protein-blocking experiment, HNE1 cells were infected with EBV, which was pre-incubated with purified NRP1^ABC^ for 1 h. For antibody against NRP2-blocking experiment, HNE1 cells were pre-incubated with an anti-NRP2 antibody (100 μg ml^−1^) or goat IgG (control) at 4 °C for 1 h and then were exposed to EBV at an MOI of 5 × 10^3^ for 3 h at 4 °C. (**d**,**e**) Overexpression of NRP1 enhanced EBV infection, while NRP2 suppressed EBV infection. HNE1 cells were transiently transfected with the expression plasmid for NRP1, NRP2 or the empty vector (pMSCV) for 24 h, followed by analysis for the expression of NRP1 and NRP2 by western blotting (**d**) or were exposed to EBV (**e**). (**f**,**g**) EGF upregulated NRP1 expression and enhanced EBV infection. HNE1 cells cultured with 10 ng ml^−1^ EGF for 24 h were analysed for the expression of NRP1 and NRP2 by western blotting (**f**) or were exposed to EBV (**g**). (**h**,**i**) EGF-enhanced EBV infection was partially dependent on NRP1. After transfected with siRNA against NRP1 for 48 h, EGF-treated HNE1 and NPEC-Bmi1 cells maintained in KSF medium supplemented with EGF were analysed for NRP1 expression by western blotting (**h**) or were exposed to EBV (**i**). For (**b**), (**c**), (**e**), (**g**) and (**i**), HNE1 or NPEC1-Bmi1 cells were exposed to EBV at an MOI of 2.5 × 10^3^ for 2 h at 37 °C, unless otherwise indicated. The percentage of GFP-positive infected cells was analysed by FACS 48 h post infection, with controls (empty vector-transfected cells or vehicle treated-cells) set to 100%. Data represent three to five independent experiments. Values in all graphs are means±s.e.m. ***P<0.001; **P<0.01; *P<0.05; Student’s *t*-test. For (**d**), (**f**) and (**h**), GAPDH served as an internal control.

To determine whether NRP1 is important for EBV infection, siRNA and blocking assays were performed. Compared with siControl-transfected HNE1 cells, siNRP1 transfectants were relatively resistant to EBV infection with an approximately twofold decrease. In contrast, knockdown of neuropilin 2 (NRP2), the homologue of NRP1, increased EBV infection by about twofold (Fig. 2a,b and Supplementary Fig. 5). Pre-incubation of EBV with the soluble ectodomain of NRP1 (NRP1^ABC^) inhibited EBV infection by about 50%, whereas an anti-NRP2 antibody increased EBV infection by about threefold (Fig. 2c).

Next, we examined the role of overexpression of NRP1 and NRP2 on EBV infection. The efficiency of EBV infection was significantly enhanced by an increase in NRP1 expression, whereas overexpression of NRP2 inhibited EBV infection (Fig. 2d,e and Supplementary Fig. 6). Epidermal growth factor (EGF) is a known cytokine that induces NRP1 expression in multiple cancer cells^29^. To examine the effect of EGF on the expression of NRP1 and EBV infection, HNE1 cells were incubated with EGF for 24 h. EGF significantly enhanced the expression of NRP1 in HNE1 cells, but did not change the level of NRP2 (Fig. 2f). Consistently, the efficiency of EBV infection was significantly enhanced by EGF (Fig. 2g, Supplementary Figs 6 and 7). In addition, EGF promotes EBV infection and the expression of NRP1 in a dose-dependent manner (Supplementary Fig. 8). Furthermore, knockdown of NRP1 led to a significantly decreased EBV infection in EGF-treated HNE1 and NPEC1-Bmi1 cells maintained in KSF medium supplemented with EGF (Fig. 2h,i and Supplementary Fig. 9), suggesting that EGF-induced uptake of EBV at least partially depended on the induction of NRP1.

NRP1 is a receptor for a number of ligands (for example, semaphorins, VEGF-A)^30^,^31^. To investigate whether the binding of NRP1 and its ligands would affect EBV infection, HNE1 cells were incubated with the indicated doses of NRP1 ligands (SEMA3A, SEMA3F or VEGFA) for 1 h before EBV infection. SEMA3A, SEMA3F or VEGFA had no effect on EBV infection (Supplementary Fig. 10); however, whether other ligands for NRP1 affect EBV infection remains to be further investigated.

### NRP1 co-localizes with EBV and binds to EBV-gB

**Figure 3:**
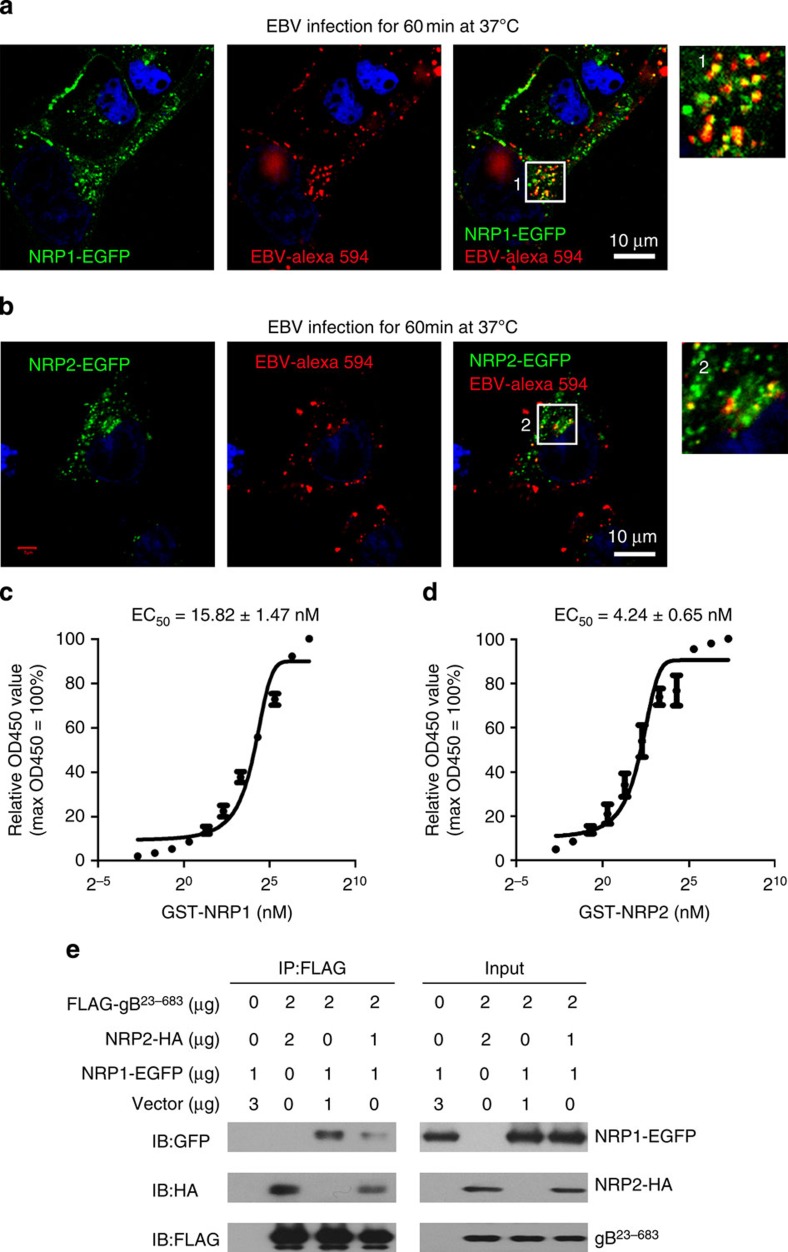
NRP1 and NRP2 co-localize with EBV and bind to EBV-gB^23–431^. (**a**,**b**) NRP1 and NRP2 co-localize with EBV. HNE1 cells transiently transfected with the indicated expression vectors for 24 h were infected with Alexa fluor 594-labelled EBV (EBV-Alexa 594). Expressions of NRP1-EGFP and NRP2-EGFP were visualized as green. EBV-Alexa fluor 594 was imaged as red. Nuclei were stained with DAPI (blue). The images of the boxed areas labelled as 1 and 2 were enlarged and shown. (**c**,**d**) The affinity constant of the interaction between gB and NRP1 or NRP2. Microtitre plates were coated with 200 ng purified FLAG-gB^23–431^ and incubated with various concentrations of GST-NRP1 or GST-NRP2, followed by the rabbit anti-GST and the HRP-conjugated anti-rabbit IgG secondary antibodies. The binding of purified EBV-gB^23–431^ and GST-NRP1 (**c**) or GST-NRP2 (**d**) were analysed by ELISA. Values are means±s.e.m. of three independent experiments. (**e**) NRP2 influences the binding of NRP1 to EBV-gB. HEK-293FT cells transfected with the indicated doses of expression vector for 36 h. The cell lysates were immunoprecipitated (IP) with an anti-FLAG antibody, followed by immunoblotting (IB) analysis with an anti-GFP, anti-HA or anti-FLAG antibody, as indicated. The experiments were performed three times with similar results.

To investigate whether NRP1 could directly mediate EBV infection, we examined the localization of EBV and NRP1 in NRP1-overexpressing HNE1 cells. Confocal microscopy revealed that both Alexa Fluor 594-labelled EBV and anti-gp350 antibody-stained EBV co-localized with NRP1-EGFP on the cell membrane or at vesicular structures (Fig. 3a and Supplementary Fig. 11). Similarly, the co-localized signals of NRP2 and EBV could also be detected (Fig. 3b).

The binding of the purified EBV gB^23–431^ and GST-NRP1 or GST-NRP2 was then analysed by ELISA. The apparent affinity constant for binding of gB and NRP2 was higher than that for gB and NRP1 (Fig. 3c,d).

As NRP1 and NRP2 played opposite effects on EBV infection, we therefore evaluated whether NRP2 would affect the binding of NRP1 to gB. Co-immunoprecipitation indicated that the interaction of NRP1 and gB was obviously reduced in the presence of NRP2 (Fig. 3e).

### NRP1 facilitates EBV internalization and fusion

**Figure 4:**
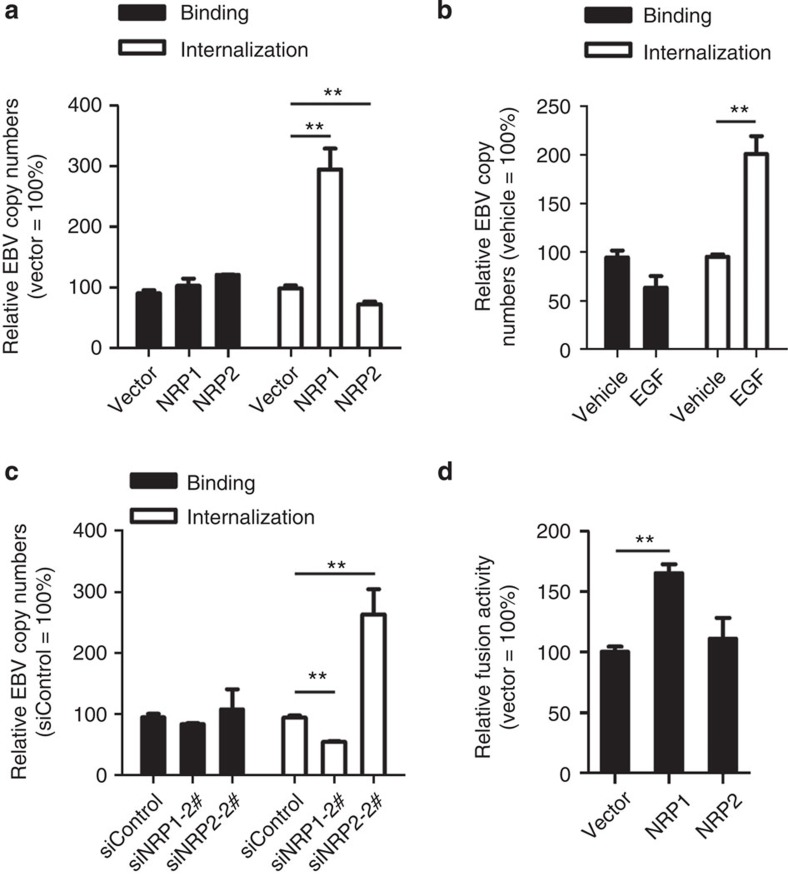
NRP1 facilitates EBV internalization and cell–cell fusion, while NRP2 inhibits EBV entry. (**a**) NRP1 promoted EBV internalization, but did not affect EBV binding. HNE1 cells transfected with overexpression plasmids for NRP1 or NPR2 were infected with EBV at 37 °C to allow virus internalization, followed by real-time PCR analysis for EBV copy number; *n*=3. (**b**) EGF-enhanced EBV internalization. EGF-treated HNE1 cells were subjected to analysis for EBV binding and internalization; *n*=6. (**c**) Downregulation of NRP1 impaired, whereas knockdown of NRP2 promoted EBV internalization in EGF-treated HNE1 cells. EGF-treated HNE1 cells transfected with siRNA against NRP1 or NRP2 were subjected to analysis for EBV binding and internalization; *n*=3. (**d**) NRP1, but not NRP2, promoted cell-cell fusion. HEK-293FT cells transfected with expression vectors for T7 polymerase, gB and gH/gL were co-cultured with HEK-293FT cells co-transfected with pT7EMCLuc, pRL-SV40, the expression plasmid for NRP1, NRP2 or the empty vector (pMSCV). The relative fusion activity was calculated as the ratio of firefly to *Renilla* luciferase activity analysed 24 h after co-culture, with the empty vector controls set to 100%; *n*=3. Graphs show mean±s.e.m. **P<0.01; Student’s *t*-test.

As mentioned above, EBV entry comprises at least two steps, including binding (attachment) and penetration (fusion)^7^. We then investigated in which step NRP1 played a role. Upregulation of NRP1 by either overexpression or EGF treatment increased EBV internalization by about 2.5-fold, while knockdown of NRP1 by siRNA caused a decrease to about 50% of the control. In contrast, overexpression of NRP2 impaired EBV internalization, while knockdown of NRP2 enhanced EBV internalization. Both gain- and loss-of-function experiments revealed that neither NRP1 nor NRP2 exerted any effect on EBV binding (Fig. 4a–c). To determine the role of NRP1 or NRP2 in the efficiency of cell-cell fusion, NRP1- or NRP2-overexpressing HEK-293FT cells were co-cultured with EBV glycoproteins (gB and gH/gL)-overexpressing HEK-293FT cells. Overexpression of NRP1 significantly promoted cell-cell fusion while overexpression of NRP2 had no effect on cell fusion (Fig. 4d).

Collectively, these data suggested that NRP1 may facilitate EBV internalization and fusion, but not binding.

### EBV enters cells via endocytosis

**Figure 5:**
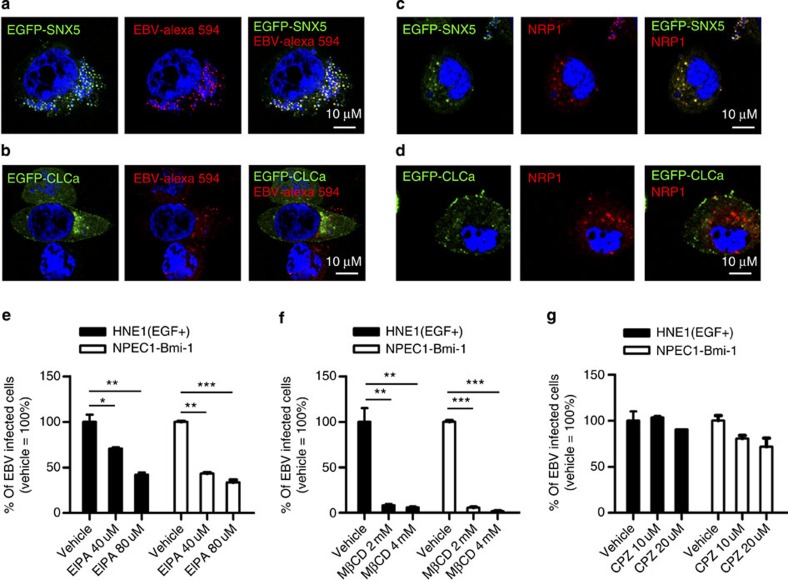
EBV enters epithelial cells via lipid raft-dependent endocytosis and macropinocytosis. (**a**,**b**) EBV co-localized with EGFP-SNX5, but not EGFP-CLCa. HNE1 cells transiently transfected with expression vectors for EGFP-SNX5 (**a**) or EGFP-CLCa (**b**) for 24 h were infected with Alexa fluor 594 labelled EBV (EBV-Alex 594) for 60 min at 37 °C. (**c**,**d**) EGFP-SNX5, but not EGFP-CLCa co-localized with NRP1. HNE1 cells transiently co-transfected with the expression vectors for NRP1-mCherry and EGFP-SNX5 (**c**) or EGFP-CLCa (**d**) for 24 h were infected with cell-free EBV. For (**a**–**d**), expressions of EGFP-SNX5 and EGFP-CLCa were visualized as green, and EBV-Alexa fluor 594 and expression of NRP1-mCherry were imaged as red. Nuclei were stained with DAPI (blue). (**e**–**g**) EBV infection was dose-dependently suppressed by EIPA (**e**) and MβCD (**f**), but not CPZ (**g**). EGF-treated HNE1 cells and NPEC1-Bmi1 cells were pre-incubated with the indicated doses of EIPA, MβCD and CPZ for 30 min, followed by EBV infection for 2 h. The cells were then washed with Hanks solution twice and cultured for 48 h until FACS analysis; *n*=3. Graphs show mean±s.e.m. ***P<0.001; **P<0.01; *P<0.05; Student’s *t*-test.

It has been reported that NRP1 mediates endocytosis via different pathways, depending on its ligands^32^. We thus examined the mechanistic basis for EBV endocytosis. Enveloped viruses penetrate into the cytosol directly through caveolae-mediated endocytosis (for particles with size of 50–80 nm), clathrin-mediated endocytosis (CME, for particles with size of 85–180 nm) and macropinocytosis (for particles with size of 0.5–10 μm)^33^. EBV is an enveloped virus with a diameter of 120–220 nm. Therefore, EBV infection is unlikely to be dependent on caveloae-mediated endocytosis. Confocal analysis showed that both EBV-Alexa fluor 594 and NRP1 co-localized with SNX5 (marker of macropinocytosis), but not with CLCa (marker of CME) (Fig. 5a–d), suggesting that NRP1-mediated EBV internalization may be dependent on macropinocytosis, but not CME. To further verify this observation, EGF-treated HNE1 cells and NPEC1-Bmi1 cells were pre-incubated with the inhibitors of macropinocytosis (5-(N-ethyl-N-isopropyl)-amiloride, EIPA), lipid raft-dependent endocytosis (Methyl-β-cyclodextrin, MβCD) and CME (Chlorpromazine, CPZ) at the indicated concentration for 30 min, and then infected with EBV for 2 h. The cells were then washed with Hanks solution and cultured for 48 h. EBV infection was dose-dependently suppressed by MβCD and EIPA, but not by CPZ in both type of cells, with preserved cell viability (Fig. 5e–g and Supplementary Fig. 12). These data demonstrated that EBV entered epithelial cells via macropinocytosis and lipid raft-dependent endocytosis, but not clathrin-mediated endocytosis.

### EBV activates NRP1-dependent EGFR signalling pathways

**Figure 6:**
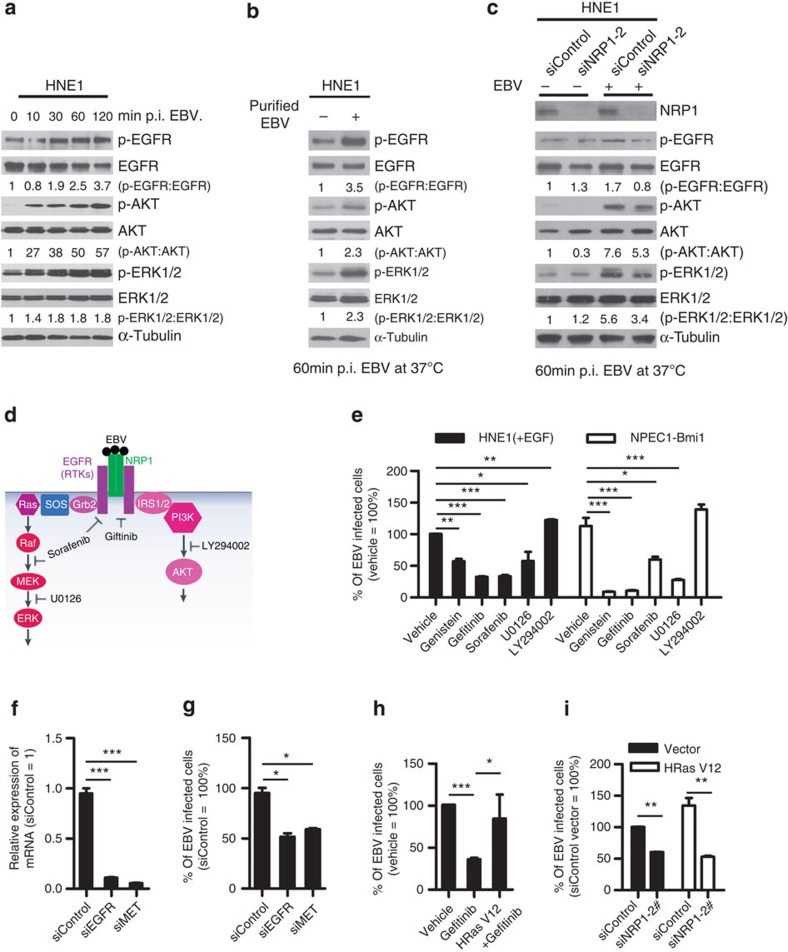
NRP1-dependent EGFR/ERK signalling pathways are activated by EBV and are also associated with the promotion of EBV infection. (**a**,**b**) EBV infection activated EGFR/AKT and EGFR/ERK pathways. Serum-starved HNE1 cells were infected with EBV (**a**) or purified EBV (**b**) at an MOI of 5 × 10^3^ for the indicated times. (**c**) EBV-activated EGFR/AKT or EGFR/ERK pathways were partially mediated by NRP1. After transfected with the indicated siRNA duplexes for 48 h, HNE1 cells were serum-starved and infected with EBV at an MOI of 5 × 10^3^ for 1 h, followed by western blotting. For **a**–**c**, the intensity of the bands, determined with the ImageJ software, was shown as indicated. α-Tubulin, internal control. (**d**) Schematic diagram of RTKs (EGFR and VEGFR2)/NRP1/Ras/ERK and RTKs/NRP1/PI3K/AKT signalling cascade inhibitors targeting the indicated members of the pathways are shown. (**e**) EGFR/Ras/ERK signalling pathway was required for EBV infection. EGF-treated HNE1 and NPEC1-Bmi1 cells pre-incubated with Gefitinib (20 μM), Sorafenib (HNE1, 20 μM; NPEC1-Bmi1, 5 μM), U0126 (50 μM) and LY294002 (50 μM) for 30 min were infected with EBV for 2 h, and then cultured in the fresh medium for 48 h until FACS analysis for the infection efficiency. (**f**,**g**) Downregulation of EGFR and c-Met impaired EBV infection. HNE1 cells were transfected with siRNA duplexes targeting EGFR or c-Met for 48 h, followed by real-time PCR analysis for the expressions of EGFR and c-Met (**f**) or FACS analysis for EBV infection efficiency (**g**). (**h**) HRas partially rescued the inhibitory effect of Gefitinib on EBV infection. HNE1 cells transfected with pMSCV-HRAS-V12 plasmid for 24 h were pre-incubated with 20 μM Gefitinib for 30 min, followed by exposure to EBV. (**i**) HRas failed to rescue the suppressive effect of silencing NRP1 on EBV infection. After transfected with siNRP1 or siControl for 36 h, HNE1 cells were reseeded, and then transfected with the plasmid for HRAS-V12 or the empty vector for 24 h, followed by exposure to EBV. The infected cells were analysed by FACS, with controls set to 100%. For (**e**–**i**), data represent three times independent experiments. Graphs show mean±s.e.m. ***P<0.001; **P<0.01; *P<0.05; Student’s *t*-test.

Macropinocytosis and lipid raft-dependent endocytosis can be induced by RTKs^34^,^35^. As a co-receptor of RTKs, NRP1 enhances the affinity of multiple growth factors, such as EGF, HGF, VEGF, PIGF and PDGF-BB, to RTKs and thus augments RTKs signalling^36^,^37^,^38^,^39^,^40^. EGFR, the prototypical RTK aberrantly expressed in NPC^41^, as well as its critical downstream signalling components AKT and ERK, was rapidly phosphorylated at 10 min post EBV infection, and the phosphorylation increased and persisted for at least 120 min (Fig. 6a). To exclude that the activation of EGFR signalling pathways was caused by the virus debris, HNE1 cells were infected with EBV purified by high-speed centrifugation in dextran T-10 density gradients for 1 h. EGFR, AKT and ERK were phosphorylated by purified EBV (Fig. 6b), indicating that the EGFR signalling pathway was indeed activated by EBV. Knockdown of NRP1 partially suppressed the phosphorylation of EGFR, AKT and ERK on EBV infection (Fig. 6c), suggesting that NRP1 was associated with EBV activation of EGFR/AKT and EGFR/ERK pathways.

To confirm the role of RTK signalling pathways in EBV infection, EGF-treated HNE1 and NPEC1-Bmi1 cells pre-incubated with inhibitors of tyrosine kinases (Genistein), MEK1/MEK2 (U0126), PI3K/AKT (LY294002), EGFR (Gefitinib) and VEGFR2/PDGFR/Raf signalling cascades (Sorafenib), were infected with EBV. Genistein, Gefitinib, Sorafenib and U0126 partially eliminated EBV entry, whereas LY294002 did not affect EBV infection (Fig. 6d,e). These data suggested that the activation of multiple RTKs and the downstream signalling Ras/Raf/MEK/ERK rather than PI3K may enhance EBV infection.

As Genistein and Sorafenib partially impaired EBV infection, we further investigated whether other signalling pathways besides EGF/EGFR were also important for EBV infection. HNE1 cells were transfected with siRNA duplexes targeting EGFR or c-Met (receptor for HGF), followed by EBV infection. The expression of EGFR and c-Met was nearly diminished in HNE1 cells transfected with siEGFR or siMET. Knockdown of either EGFR or c-Met impaired EBV infection by about 50% (Fig. 6f,g), indicating that there may be indeed other RTKs contributing to EBV infection.

Furthermore, activated Ras (HRas V12) partially rescued the suppressive effect of Gefitinib on EBV infection, confirming that HRas mediates EGFR-dependent EBV entry (Fig. 6h). Knockdown of NRP1 even suppressed EBV infection in HNE1 cells with persistently activated Ras (Fig. 6i), suggesting that the activated Ras signalling was associated with but insufficient for EBV infection, and highlights the role of NRP1 in facilitating EBV entry into nasopharyngeal epithelial cells.

## Discussion

Epstein–Barr virus (EBV), an ubiquitous human herpesvirus, has been classified as a group 1 carcinogen^42^,^43^. It is aetiologically associated with lymphoid and epithelial tumours, suggesting its primary tropism for these cells. The mechanism contributing to EBV infection of B cells has been well documented, while the mechanisms of cell-free EBV infection of epithelial cells remain elusive. Here, we established a cell model relatively susceptible to cell-free EBV infection, and highlighted the important role of NRP1 in mediating cell-free EBV infection of nasopharyngeal epithelial cell lines and EBV activated the RTK signalling pathway.

In addition to cell-free EBV infection, cell-to-cell contact is supposed to be an efficient mode of EBV infection of diverse human epithelial cells^28^. Like cell-free EBV infection, EGF promotes the transmission of EBV from infected Akata cells to uninfected HNE1 cells, partially depending on the expression of NRP1 on the host cells (Supplementary Fig. 13), suggesting an important role of NRP1 and EGF in facilitating not only cell-free EBV infection, but also cell-to-cell contact-mediated infection.

EBV gB, the most highly conserved glycoprotein, is necessary for EBV infection. It mainly presented as the trimeric form, consistently with the previous report about the crystal analysis of EBV gB. EBV gB^23–431^, gB^23–683^ (the ectodomain of EBV-gB) and gB^23–853^ (the almost full-length EBV-gB) showed interaction with NRP1. In addition to the CendR motif, gB^23–88^ is another important element contributing to the interaction between NRP1 and gB. Reimer *et al*.^24^ revealed that, although linker insertions at position 88 did not affect cell-surface expression of gB, it abrogated the ability of the variant protein to mediate fusion. Backovic *et al*.^23^ reported residues 88 was a hydrophilic residue. Therefore, gB^23–88^ may define novel binding sites for ligands, such as a gB receptor or other EBV envelope glycoproteins involved in EBV infection^23^. We demonstrated that NRP1 and NRP2 interacted with the glycoprotein gB, but had opposite effect on EBV infection. Overexpression of NRP1 significantly promoted cell-cell fusion, while overexpression of NRP2 had no effect on cell fusion. Both being as co-receptors for RTKs, NRP1 and NRP2 bind to different ligands. NRP1 binds to Sema3A and initiates plexin signalling, which activates CRMP, ERK and Rac1, whereas NRP2 binds to Sema3F and activates Rac GTPase-activating protein (GAP) β2-Chimaerin^30^,^44^,^45^. Although the exact mechanism underlying the discrepancy of NRP1 and NRP2 on the susceptibility of EBV infection remains to be determined, it could be attributed to the different ligands they bind, and the distinct downstream signalling pathways they activated. However, we found that the ligands for NRP1 (SEMA3A, SEMA3F and VEGF-A) had no effect on EBV infection, whether other ligands for NRPs affect EBV infection of nasopharyngeal epithelial cells remain further investigation.

It has been reported that the interaction between epithelial integrins (for example, αvβ6, αvβ8 or α5β1) and EBV envelope proteins (gHgL or BMRF2) is required for EBV infection of epithelial cells originated from tongue, nasopharyngeal and gastric carcinoma^46^,^47^. We found integrins αv,β1 and β6, but not integrins α5 and β8, may contribute to EBV infection of nasopharyngeal epithelial cells (Supplementary Fig. 14).

Integrins bind the RGD/KGD motif-containing peptide, which is cleaved by cell surface-associated proteases to expose the cryptic CendR element, RXXK/R, at the C terminus. The CendR element then mediates binding to NRP1, resulting in the penetration of cells and tissues^18^. Both the full-length and cleaved forms of gB are present in the purified EBV (Supplementary Fig. 2). We therefore suppose the model that EBV entry into epithelial cells: for the full-length gB-containing EBV, EBV binds to nasopharyngeal epithelial cells through the interaction between epithelial integrins (αv, β1 and β6), or other unknown factors and envelope proteins (gHgL or BMRF2). Then, EBV gB was cleaved by furin to expose the CendR motif. For cleaved gB containing EBV, the CendR motif may be already exposed^16^,^23^. Followed by the interaction between the CendR motif on the cleaved EBV gB and NRP1, EBV enters into epithelial cells.

In addition to Gefitinib, Sorafenib and Genistein partially impaired EBV infection, suggesting there are multiple RTKs and the downstream signalling pathways other than EGFR contributing to EBV infection. This point was further confirmed by the evidence that knockdown of c-Met attenuated EBV infection. Therefore, various RTKs signalling pathway may promote EBV infection, and the detailed mechanisms are deserved to be further investigated.

Multiple viruses, such as HTLV-1, possess CendR motifs within their capsid proteins. Similarly, NRP1 also serves as an entry factor for HTLV-1 through interaction with the KPXR element (a CendR motif) exposed on its surface subunit (SU). Whether NRP1-mediating RTK signalling pathways also play important role in HTLV-1 infection remains to be further investigated.

**Figure 7:**
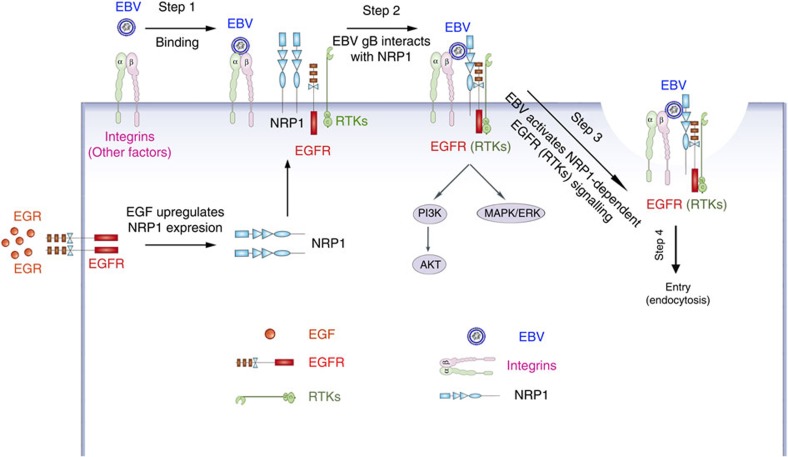
Schematic showing the potential role of NRP1 in mediating EBV entry and EBV-activated signalling pathways. Step 1: EBV binds to the nasopharyngeal epithelial cells through the interaction between integrins (αv, β1 and β6) and EBV envelope proteins (gHgL or BMRF2). Step 2: EBV gB interacts with NRP1, which recruits RTKs (such as EGFR). The expression of NRP1 is enhanced by EGF treatment. Step 3: EBV activates EGFR/AKT and EGFR/ERK pathways, which partially depend on NRP1. Step 4: EBV enters epithelial cells via macropinocytosis and lipid raft-dependent endocytosis. Together, such complex signalling events trigger EBV entry into nasopharyngeal epithelial cells.

EBV gB is critical to mediate virus cell fusion^48^,^49^. We found that NRP1 serves as an entry factor for EBV infection of nasopharyngeal epithelial cells. However, whether NRP1 also serves a similar function for EBV entry into B cells has not been explored and deserves further investigation, although the expression of NRP1 on B cells is quite low (Supplementary Fig. 15). NRP1 not only functioned as an entry factor for EBV to entry into epithelial cells, but was also associated with EBV-activated EGFR/RAS/ERK signalling, which in turn potentiated EBV infection (Fig. 7). As multiple inhibitors of receptor tyrosine kinase signalling pathways impaired EBV infection, further investigations are required both to investigate whether receptor tyrosine kinases beyond EGFR are also involved in promoting EBV infection, and to elucidate the detailed mechanism by which NRP1 contributes to viral entry. Such findings would assist in the development of anti-EBV agents that target this crucial stage in virus infection.

## Methods

### Reagents

The reagents used were as follows: mouse monoclonal antibodies against FLAG (F1804, Sigma-Aldrich, 1:2,000 dilution), α-tubulin (T6074, Sigma-Aldrich, 1:2,000 dilution), GAPDH (KC-5G4, Kangcheng Biotech., 1:3,000 dilution), GFP (sc-9996, Santa Cruz, 1:3,000 dilution) and EBV-gp350 (72A1, 1:200 dilution); rabbit monoclonal antibodies against NRP1 (2621-S, Epitomics Inc, 1:1,000 dilution), phospho-AKT1-Ser473 (#4060, Cell Signaling Technology, CST, 1:1,000 dilution), p-EGFR (Tyr1068, #3777, CST, 1:1,000 dilution), EGFR (#4267, CST, 1:1,000 dilution), phospho-ERK1/2 (Thr202/Tyr204, #4376, CST, 1:1,000 dilution); rabbit polyclonal antibodies against AKT (KC-5A01, Kangcheng Biotech., 1:1,000 dilution), ERK1/2 (KC-5E01, Kangcheng Biotech., 1:1,000 dilution); sheep polyclonal antibody against NRP1 (AF3870, R&D Systems, 1:1,000 dilution); goat polyclonal antibody against NRP2 (AF2215, R&D Systems, 1:500 dilution); normal mouse/rabbit/goat IgG (R&D Systems). The horseradish peroxidase (HRP)-conjugated goat-anti-mouse/rabbit secondary antibodies (Fisher Scientific, 1:3,000 dilution). HRP-conjugated donkey-anti-sheep secondary antibody (1:2,000 dilution) was gifted from R&D Systems. Alexa Fluor 594-conjugated goat-anti-mouse (Molecular Probes, 1:2,000 dilution); 4′-6′-diamidino-2-phenylindole (DAPI; Molecular Probes); soluble NRP1 (sNRP1, R&D Systems); EGF (E9644, Sigma-Aldrich); BSA (A4737, Sigma-Aldrich). Gefitinib and Sorafenib were purchased from SELLECK; U0126 and LY294002 were purchased from Merck; Genistein, CPZ (Chlorpromazine), MβCD (Methyl-β-cyclodextrin) and EIPA (5-(N-ethyl-N-isopropyl)-amiloride) were purchased from Sigma-Aldrich. All other reagents were obtained from Sigma-Aldrich, unless otherwise indicated.

### Cell culture

NPECs-Bmi1 cells and NP69 cells, grown in keratinocyte/serum-free (KSF) medium (Invitrogen), were immortalized nasopharyngeal epithelial cells induced by Bmi-1 or SV40T, respectively. All human EBV-negative NPC cell lines (SUNE1, SUNE2, 6-10B, CNE1, CNE2 and HNE1) and EBV-positive cells (Akata and Namalwa) maintained in our laboratory were cultured in RPMI 1640 medium (GIBCO) supplemented with 5% fetal bovine serum (FBS, GIBCO). HEK-293FT cells were cultured in Dulbecco’s modified Eagle’s medium (DMEM, Life Technologies) supplemented with 10% FBS. Cells were grown in a humidified 5% CO_2_ incubator at 37 °C and passaged using standard cell culture techniques.

Primary nasopharyngeal epithelial cells (NPEC03 and NPECw) were established as followings: fresh biopsies of the nasopharynx, which were pathologically exclusive of NPC, were collected from the Department of Head and Neck Surgery at Sun Yat-Sen Memorial Hospital, Sun Yat-Sen University. The specimen was cut into pieces and plated onto T-25 culture flask (Falcon) containing 2 ml of KSF medium. The cells grown out from the biopsies were propagated and stained with anti-keratin (ZM-0069, Zhongshan Golden Bridge Biotechnology). This study was approved by the Institute Research Ethics Committee at the Sun Yat-Sen Memorial Hospital. Written informed consent was obtained from each patient.

### Plasmids

To construct expression vectors for NRP1 and NRP2, the full-length complementary DNA (cDNA) sequences of NRP1 and NRP2 were PCR-amplified using cDNA from NPEC1-Bmi1 cells, and then integrated into the XhoI/EcoRI sites in pMSCV-puro vector (Clontech) and into the HindIII/SalI sites in pLncx2-neo vector (Clontech), named as pMSCV-NRP1 and pLncx2-NRP2, respectively.

To obtain the soluble form of the extracellular ABC domain of NRP1 or NRP2, NRP1^30–862^ amplified from pMSCV-NRP1 and NRP2^29-852^ amplified from pLncx2-NRP2 were inserted into pET32a vector (Merck) and pGEX-6p-1 vector (Invitrogen) to generate pET32a-NRP1^ABC^, pGEX-NRP1^ABC^ and pGEX-NRP2^ABC^, respectively.

To generate the expression vector (pCDNA3.1(+)-NRP1) for soluble NRP1 used in an *in vitro* transcription–translation system, NRP1^30-636^ was PCR-amplified from pMSCV-NRP1, and then inserted into pCDNA3.1(+) vector (Invitrogen).

To determine the fusion efficiency, plasmids pCAGT7 encoding T7 RNA polymerase, and pT7EMCLuc carrying the firefly luciferase gene under the control of the T7 promoter (gifted from Professor R. Longnecker, Northwestern University) were used.

To investigate whether gB interacted with NRP1, the plasmids (pCEP-his-FLAG-gB^23–427^, pCEP-his-FLAG-gB^23–431^, pCEP-his-FLAG-gB^23–683^, pCEP-his-FLAG-gB^89–431^ and pCEP-his-FLAG-gB^89–427^) were constructed. The fragments gB^23–427^, gB^23–431^, gB^23–683^, gB^89–431^ and gB^89–427^ were PCR-amplified from the EBV glycoprotein gB expression plasmid p2670 (gifted from Professor W. Hammerschmidt, Department of Gene Vectors, Helmholtz Zentrum München) and cloned into the pCEP-his vector.

To study the co-localization of EBV, NRP1 and the endocytosis markers, fluorescent protein-tagged NRP1 expression vectors (pEGFP-NRP1 and pMSCV-NRP1-Mcherry) were generated. The cDNA sequence of NRP1 was PCR-amplified from the vector pMSCV-NRP1, and then integrated into EcoRI/BamHI upstream of the start codon of EGFP expression cassette in the pEGFP-N1 vector (Clontech), under the control of CMV promoter, naming as pEGFP-NRP1, or inserted into XhoI/EcoRI sites upstream of the start codon of Mcherry gene (amplified from pCDNA-Mcherry gifted from Professor Jun Li, Sun Yat-Sen University) in a modified pMSCV vector, under the control of PGK promoter, naming as pMSCV-NRP1-Mcherry. The full-length cDNA sequence of SNX5 was PCR-amplified using the cDNA from NPEC1-Bmi1 cells and cloned into the pEGFP-C2 vector (Clontech), naming as pEGFP-SNX5. Plasmid EGFP-CLCa1 encoding EGFP-tagged *CLCa1* gene was gifted from Professor Yoshihiro Kawaoka (University of Wisconsin-Madison). Plasmid pMSCV-HRAS-V12 for the activated Ras was gifted from Professor Vimla Band (the University of Nebraska Medical Center). Primers for cloning are listed in Supplementary Table 1.

### siRNA oligoribonucleotides

ON-TARGET plus SMART pool siRNA duplexes targeting ITGA5 (Cat# M-008003-02), ITGAV (Cat# M-004565-03), ITGB1 (Cat# M-004506-00), ITGB6 (Cat# M-008012-01), ITGB8 (Cat# M-008014-02) and the negative control siRNA (siControl, Cat# D-001220-01-20) duplex were purchased from Dharmacon (Rockford). The negative control RNA duplex (siControl) for siRNA was scrambled siRNA. The siRNA duplexes targeting two distinct sites of human *NRP1* mRNA (NCBI, NM_003873.5, Gene ID: 8829) were denoted as siNRP1-1# and siNRP1-2#, while the siRNAs duplexes targeting two distinct sites of human *NRP2* mRNA (NCBI, NM_003872.2, Gene ID: 8828) were named as siNRP2-1# and siNRP2-2#. The siRNA duplexes against NRP1 and NRP2 were synthesized by GenePharma (Shanghai). All siRNA duplexes are listed in Supplementary Table 2.

### Cell transfection

Cell transfection was performed with Fugene HD (Roche), Lipofectamine 2000 (Invitrogen) or Lipofectamine RNAiMAX (Invitrogen) as indicated, according to the manufacturer’s instructions. For overexpression experiments, HNE1 cells were plated at a density of 4–6 × 10^4^ cells per well in 24-well plates. Sixteen hours after seeding, cells were grown to about 40% confluence, and each well received 1.5 μl Fugene HD and 0.75 μg of the indicated plasmids. For immunoprecipitation assay, HEK-293FT cells were plated in six-well plates at a density of 4 × 10^5^ cells per well and grown to about 80% confluence. Sixteen hours after seeding, each well was co-transfected with 1 μg of pCEP-his-FLAG-gB^23-431^ and 1 μg of pEGFP-NRP1 or pEGFP-NRP2 with 5 μl Lipofectamine 2000 for 36 h. For siRNA experiments, a final concentration of 50 nM siRNA duplex was reversely transfected with Lipofectamine RNAiMAX, unless otherwise indicated.

### Virus production

Akata cells carrying EBV, in which the thymidine kinase gene was interrupted with a double cassette expressing GFP and neomycin resistance gene, were resuspended in FBS-free RPMI 1640 medium at a concentration of 2–3 × 10^6^ cells per ml, followed by induction with 0.75% (v/v) of goat anti-human immunoglobulin G serum (Shuangliu Zhenglong Biochem.Lab) for 6 h at 37 °C. After culture in fresh RPMI1640 medium supplemented with 4% FBS for 3 days, virus from the supernatant was collected under sterile conditions, passed through two Millipore filters (0.8 and 0.45 μm), 100-fold concentrated by high-speed centrifugation with 50,000 *g*, and then resuspended in fresh FBS-free RPMI1640 (ref. 50). The virus was stored at −80 °C and thawed immediately before infection.

### Cell-free EBV infection

EBV-negative NPEC/NPC cells were plated at a density of 4–6 × 10^4^ cells per well in 24-well plates and grown to about 60% confluence. Cells were briefly washed with Hanks solution twice and were infected with 200 μl EBV at a multiplicity of infection (MOI) of about 2.5 × 10^3^ for 3 h at 37 °C, unless indicated. After brief wash with Hanks solution twice to remove unbound virus, cells were cultured in the fresh medium for 48 h. The GFP-positive infected cells were determined by fluorescence microscopy (Olympus) and/or flow cytometry (Beckman Coulter FC500) at the indicated times post infection.

### Determination of MOI

To determine the MOI (a multiplicity of infection) of EBV, TaqMan real-time PCR was used to detect the BamHI-W fragment region of the EBV genome^51^. A calibration curve was performed, using DNA extracted from the EBV-positive cell line Namalwa, which contains two integrated viral genomes per cell, as a standard.

To evaluate the infection efficiency of the newly purified EBV, 1 × 10^5^ HNE1 cells plated in 24-well plate were infected with serial dilutions of EBV. The percentage of infected cells was analysed by flow cytometry 48 h post infection. EBV infection increased along with the virus titres. About the MOI of 2 × 10^3^ was required for 20–30% of HNE1 cells to be infected, while an MOI of 1 × 10^4^ was required for >80% of HNE1 cells to be infected. Therefore, EBV infection of epithelial cells was mediated in a virus titer-dependent manner.

### Virus binding and internalization

Cells were seeded at the density of 5 × 10^4^ cells per well in 24-well plates for 24 h. To measure virus binding, cells were washed with ice-cold Hanks solution twice and then were incubated with EBV for 3 h at 4 °C to allow cell surface binding. The cells were then washed three times with ice-cold Hanks solution to remove unbound virus. To measure virus internalization, cells were washed with Hanks solution twice and then were incubated with EBV for 2 h at 37 °C to allow internalization, then washed three times with Hanks solution to remove unbound virus. To remove the surface-bound virus, the cell was resuspended in 200 μl trypsin-EDTA (GIBCO) and proteinase K for 5 min, and the cell pellets were then washed three times with Hanks solution.

### Cell fusion assay

HEK-293FT cells co-transfected with the expression vectors (pCAGT7, p2670, pCAGGS-gH and pCAGGS-gL; expressing T7 polymerase, gB, gH or gL, respectively) were used as effector cells. HEK-293FT cells co-transfected with pMSCV-NRP1 (or the empty vector pMSCV as control), a reporter plasmid pT7EMCLuc encoding the luciferase gene driven by the T7 polymerase and an internal control plasmid pRL-SV40 encoding the *Renilla* luciferase gene driven by the SV40 promoter served as target cells. Twenty-four hours post transfection, the effector and target cells were detached using trypsin-EDTA and co-cultured in a 24-well plate at a density of 2 × 10^5^ for 24 h. Firefly and *Renilla* luciferase activities were assayed by using the dual-luciferase reporter assay system (Promega) with the Veritas luminometer (Promega). The relative fusion activity was calculated as the ratio of firefly luciferase activity to *Renilla* luciferase activity. The mean value for pMSCV vector-transfected cells was normalized to 100% relative fusion activity.

### Preparation of recombinant soluble NRPs protein

For blocking assays, soluble NRP1^ABC^ protein was prepared. The plasmid (pET32a-NRP1^ABC^), expressing the soluble NRP1^ABC^, was transformed into *E. coli* strain BL21 (DE3). A single colony was inoculated into 5 ml LB-Amp (LB medium containing 100 μg ml^−1^ ampicillin) and grown at 37 °C with vigorous shaking. The seed culture was then inoculated into 500 ml LB-Amp medium. When the OD600 reached 0.6–0.7, induction was initiated by the addition of 50 μM isopropyl-1-thio-β-D-galactopyranoside (IPTG), followed by a further 3 h shaking before collection. The culture was then centrifuged, resuspended in 50 ml cold NTA-buffer (50 mM sodium phosphate at pH 8.0 and 0.3 M NaCl) containing 1 mM PMSF, and then sonicated on ice and clarified by centrifugation at 12,000 *g* for 10 min. The 6 × His-FLAG-tagged NRP1^ABC^ protein in the precipitate was purified by Ni-NTA agarose (Fisher Scientific). After elution with buffer (50 mM sodium phosphate at pH 7.0, 300 mM NaCl, 6 M urea and 150 mM imidazole), soluble NRP1^ABC^ protein was extensively dialysed with RPMI1640, concentrated with Amicon Ultra-4 (Millipore) centrifugation tubes with a 10-kDa molecular mass cutoff and was stored at −20 °C.

To determine the affinity constant, recombinant soluble GST-NRP1^ABC^ and GST-NRP2^ABC^ proteins were prepared. The plasmid pGEX-GST-NRP1^ABC^ or pGEX-GST-NRP2^ABC^, expressing soluble GST-NRP1^ABC^ or GST-NRP2^ABC^, was transformed into *E. coli* strain Rosetta. A single colony was grown in the LB-Amp medium. When the OD600 reached 0.6–0.7, induction was initiated by 200 μM IPTG, followed by an overnight shaking at 18 °C before collection. The culture was centrifuged and homogenized with high pressure on ice and then centrifuged at 50,000 *g* for 120 min. The GST-NRP1^ABC^ and GST-NRP2^ABC^ proteins were purified by Glutathione agarose (Fisher Scientific). After elution with 10 mM GSH buffer (50 mM Tris-HCl, pH 7.5, 150 mM NaCl and 10 mM GSH), the protein was extensively dialysed with PBS buffer, and then stored at −20 °C.

### Determination of affinity constant by ELISA

Microtiter plates were coated with 200 ng Flag-gB overnight at 4 °C in a 96-well plate, followed by blocking with 5% BSA in PBS for 2 h at RT, and then incubated with various concentrations of GST-NRP1 and GST-NRP2 proteins for 2 h at RT. After washing with PBST (0.05% Tween-20 in PBS), the plate was incubated with a Rabbit anti-GST antibody (1:4,000 dilution) for 2 h at RT. The plate was then washed and incubated at 37 °C for 1 h with HRP-conjugated Goat anti-Rabbit antibody. After addition of the substrate tetramethyl-benzidine, absorbance was measured at a wavelength of 450 nm using the Spectramax M5 (Molecular Devices).

### Blocking assays

Blocking assays were performed with soluble NRP1^ABC^ or antibody against NRP2. To investigate whether soluble NRP1^ABC^ would block EBV infection, EBV pre-incubated with soluble NRP1^ABC^ or BSA (as a negative control) at 250 μg ml^−1^ for 1 h, infected of HNE1 cells for 2 h at 37 °C. To determine whether antibody against NRP2 would promote EBV infection, HNE1 cells were pre-incubated with 20 μg anti-NRP2 antibody or goat IgG (as a negative control) for 1 h and then were infected with EBV in the presence of an anti-NRP2 antibody (100 μg ml^−1^) for 3 h at 4 °C. Cells were collected at 48 h post infection. The percentages of GFP-positive infected cells were determined by a fluorescence microscope and/or flow cytometry analysis.

### Immunoprecipitation

The transfected cells were lysed in radioimmunoprecipitation assay (RIPA) buffer (20 mM Tris-HCl, pH 7.5, 150 mM NaCl, 2.5 mM MgCl_2_, 5% glycerol and 0.5% Triton X-100) containing 1 mM phenylmethylsulfonyl fluoride (PMSF) and Roche Complete protease inhibitor cocktail (Roche Diagnostics Ltd, Mannheim, Germany). After centrifuged at 15,000 *g* for 20 min at 4 °C, the lysate was preincubated with protein G-Sepharose beads (GE Healthcare) to preclear and then centrifuged at 15,000 *g* for 5 min to remove the beads. The supernatant was incubated with 2.5 μg (5 μg ml^−1^) of the indicated antibody at 4 °C overnight, and the immune complexes were captured by protein G-Sepharose beads for 2 h at 4 °C. After washing three times in RIPA buffer and twice in PBS to remove unbound proteins, the sample was suspended in 2 × SDS-sample buffer and boiled for 5 min. The complex proteins were then analysed by western blotting, using specific detection antibodies.

### Pull-down assay

Soluble NRP1^30-636^ (sNRP1^30-636^) was expressed by TnT Quick Coupled Transcription/Translation Systems (promega), in the presence of canine pancreatic microsomal membranes, according to the manufacturer’s instructions. In brief, for each reaction, 1 μg of pCDNA3.1(+)-sNRP1 and 1.5 μl canine pancreatic microsomal membranes were incubated for 60 min at 30 °C.

HEK-293FT cells were plated in 10-cm^2^ dish at a density of 3–4 × 10^6^ cells per well and grown to 60–70% confluency. After 16 h, each dish was transfected with 20 μg of pCEP-his-FLAG-gB^23-431^, pCEP-his-FLAG-gB^24-427^, pCEP-his-FLAG-gB^89-431^ or pCEP-his-FLAG-gB^89-427^ with calcium phosphate transfection for 36 h, followed by immunoprecipitation with anti-FLAG (M2) Sepharose beads (Sigma). The M2-precipitated products were then incubated with sNRP1^30-636^ overnight at 4 °C. After washing three times in RIPA buffer to remove unbound proteins, the sample was suspended in 2 × SDS-sample buffer and then analysed by western blotting.

### EBV labelling with Alexa Fluor 594

Labelling of EBV was performed with Alexa Fluro 594 (Aime-Reactive probes; MP) according to the manufacturer’s instructions and published procedures^52^,^53^. In brief, 20 μl of 5 mg ml^−1^ Alexa Fluor 594 dissolved in dimethyl sulfoxide (DMSO) was mixed with 2 ml of 200-fold concentrated EBV in FBS-free RPMI1640 and 200 μl of 0.5 M carbonate-bicarbonate buffer at pH 9.0 for 1 h in the dark at room temperature. To separate the labelled EBV from the free dye, labelled EBV was diluted 10 times with cold RPMI1640, centrifuged at 50,000 × g for 90 min at 4 °C, and then resuspended in 1.5 ml fresh FBS-free RPMI1640. The concentrated labelled virus was purified in dextran T-10 gradients, followed by re-suspension in 2 ml of fresh FBS-free RPMI1640. The purified Alexa Fluro 594-labelled EBV was subsequently dialysed four times with 500 ml RPMI1640 per time, and then stored at −80 °C until use.

### Immunofluorescence confocal microscopy

HNE1 cells were seeded on coverslips in 24-well plates at a density of 5 × 10^4^ cells per well for 12–16 h, and then transfected with the indicated plasmids for 24 h. Transfected cells infected with the unlabelled EBV at an MOI of 5 × 10^3^ for 1 h at 37 °C were briefly washed with PBS twice, fixed with 3% paraformaldehyde in PBS for 20 min, and then permeabilized with 0.1% Trtion X100 in PBS for 5 min. After blocking with 5% BSA in PBS, EBV-infected cells were stained with an antibody against gp350 (72A1, 1:200 dilution) for 4 °C overnight, and washed with PBST three times, followed by incubation with Alexa Fluor 594-labelled goat antibody to mouse IgG (1:2,000 dilution). After wash with PBST three times, cells were mounted with ProLong Gold mounting medium (Invitrogen) containing 0.2 μg ml^−1^ DAPI, which stains nuclei. Transfected cells infected with Alexa Fluor 594-labelled EBV were washed, fixed and then mounted in mounting medium. The confocal images were acquired using a Leica TCS SP5 confocal laser scanning microscope.

### Western blotting

Western blotting analysis was performed as previously described^54^. In brief, cells were lysed in RIPA buffer containing a protease inhibitor mixture (Roche) and incubated on a rocker at 4 °C for 15 min. The protein concentration of the lysates were measured using the BCA protein assay kit (Pierce) and were normalized to equal amounts of protein, separated by 9% SDS/PAGE, transferred to PVDF and probed with the indicated primary antibodies. After probed with the indicated antibodies, the blot was incubated with species-specific HRP-conjugated secondary antibodies, and the immunoreactive bands were visualized by enhanced chemiluminescence (ECL, Pierce). The same membranes were then stripped and reprobed with mouse monoclonal antibodies against GAPDH or α-tubulin to confirm equal loading of the samples. Full scans of all western blots are included in Supplementary Fig. 16–19.

### RNA extraction, EBV DNA extraction and real-time PCR

Total cellular RNA was extracted from cultured cells using the TRIzol reagent (Invitrogen) according to the manufacturer’s instructions. cDNA was synthesized from 2 μg of the total RNA using a reverse transcriptase kit (Invitrogen). The mRNA level was evaluated by qRT–PCR using the Power SYBR Green qPCR SuperMix-UDG (Invitrogen) and was analysed on Roche Lightcycler 480. All the gene expressions were normalized to the housekeeping gene *GAPDH*, used as an internal standard. Primers are listed in Supplementary Table 3. EBV DNA was extracted from EBV-infected cells using Omega tissue DNA Mini Kit (Omega) as recommended by the manufacturer. The copy number of EBV bound to the cell surface or internalized into HNE1 cells was measured using TaqMan real time PCR for detection of the BamHI-W fragment region of the EBV genome. Real-time PCR for the *GAPDH* DNA was used for cell counting estimation. A calibration curve was performed with each analysis, using DNA extracted from the EBV-positive cell line Namalwa, which contains two integrated viral genomes per cell, as a standard. The EBV copy number was expressed as a ratio of the copy number of the EBV genome to the copy number of the *GAPDH* DNA.

### Statistical analyses

The data are expressed as the mean±s.e.m. from at least three independent experiments. Statistical analyses were performed with Graphpad Prism 5 (GraphPad Software, San Diego, CA, USA).

## Author contributions

H.-B.W. and M.-S.Z. designed the study. H.-B.W and H.Z. performed the key experiments and analysed the data. Y.D. and Y.L. performed real-time PCR experiments, pull-down assay and cell culture. G.-K.F., B.Z., D.X., Q.Z., W.-L.L. provided helpful comments. H.-B.W. and J.-P.Z. prepared figures and graphs. H.D. prepared the recombinant protein. M.-Z.L assisted with cell culture. W.-L.H, S.W.T., L.H.F., Y.-X.Z. and E.K. contributed key reagents and provided import comments for the study design. H.-B.W., J.-P.Z. and M.-S.Z. wrote the manuscript. M.-S.Z. supervised the project.

## Additional information

**How to cite this article:** Wang, H.-B. *et al*. Neuropilin 1 is an entry factor that promotes EBV infection of nasopharyngeal epithelial cells. *Nat. Commun.* 6:6240 doi: 10.1038/ncomms7240 (2015).

## Supplementary Material

Supplementary Information Supplementary Figures 1-19 and Supplementary Tables 1-3
