# Supplementary material for: Neuropilin 1 is an entry factor that promotes EBV infection of nasopharyngeal epithelial cells
Source: Nat Commun. 2015 Feb 11;6:6240. doi: 10.1038/ncomms7240 (PMC4339892; doi:10.1038/ncomms7240)
Supplement: Supplementary Information — Supplementary Figures 1-19 and Supplementary Tables 1-3 [file ncomms7240-s1.pdf]

Supplementary Information

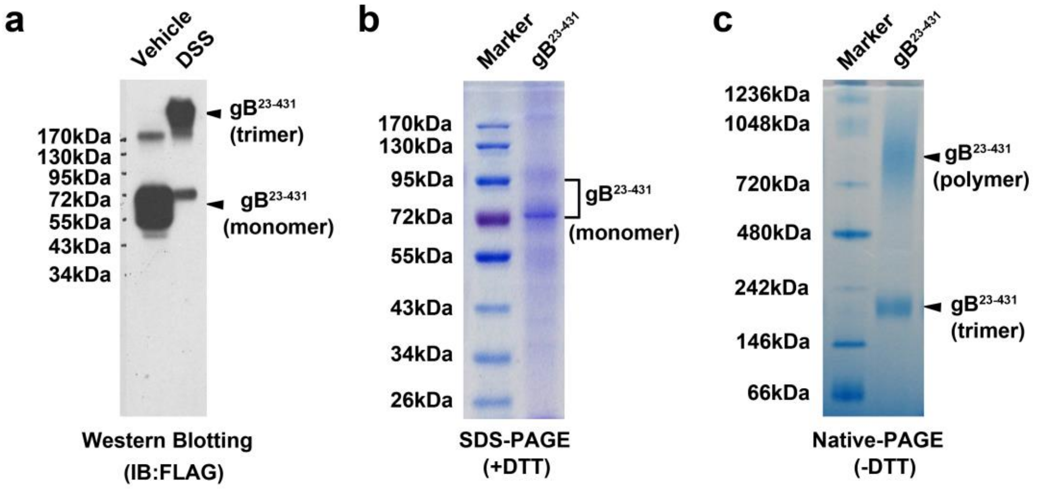

Supplementary Figure 1. EBV-gB$^{23\text{-}431}$ mainly exists as trimer in HEK 293FT cells.

(**a**) Western blotting analysis for DSS crosslinked FLAG-gB$^{23\text{-}431}$. HEK 293FT cells transfected with pCEP-FLAG-gB$^{23\text{-}431}$ for 36h were crosslinked with 0.5 mg ml^{-1} DSS (disuccinimidyl suberate) or the vehicle (DMSO, as the control) and then analyzed by immunoblotting (IB) with FLAG. DSS-cross-linked bands were seen at larger than 170 kDa. (**b**) SDS-PAGE analysis for purified EBV-gB$^{23\text{-}431}$. FLAG-tagged EBV-gB$^{23\text{-}431}$, purified with a tandem affinity-purification approach from the supernatant of HEK293FT-pCEP-FLAG-gB$^{23\text{-}431}$ stable cell line, was analyzed by SDS-PAGE. The bands with molecular weight of about 72 kDa and 96 kDa were supposed as monomeric FLAG-tagged EBV-gB$^{23\text{-}431}$. (**c**) Native-PAGE analysis for purified EBV-gB$^{23\text{-}431}$. The bands with molecular weight of around 240 kDa and 720 kDa were supposed as the trimer and mutimeric forms of FLAG-tagged EBV-gB$^{23\text{-}431}$, respectively.

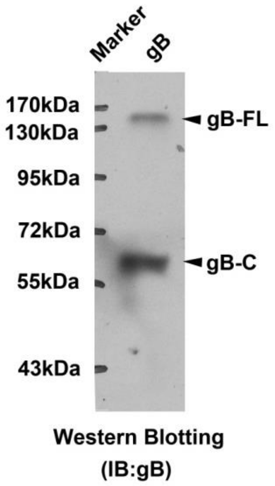

Supplementary Figure 2. Western blotting analysis for the expression of gB in the purified EBV. EBV was concentrated by high speed centrifugation with 50,000g and purified by dextran T-10 density gradients, and then subjected to western blotting analysis for the presence of gB. gB-C, cleaved gB; gB-FL, full-length gB.

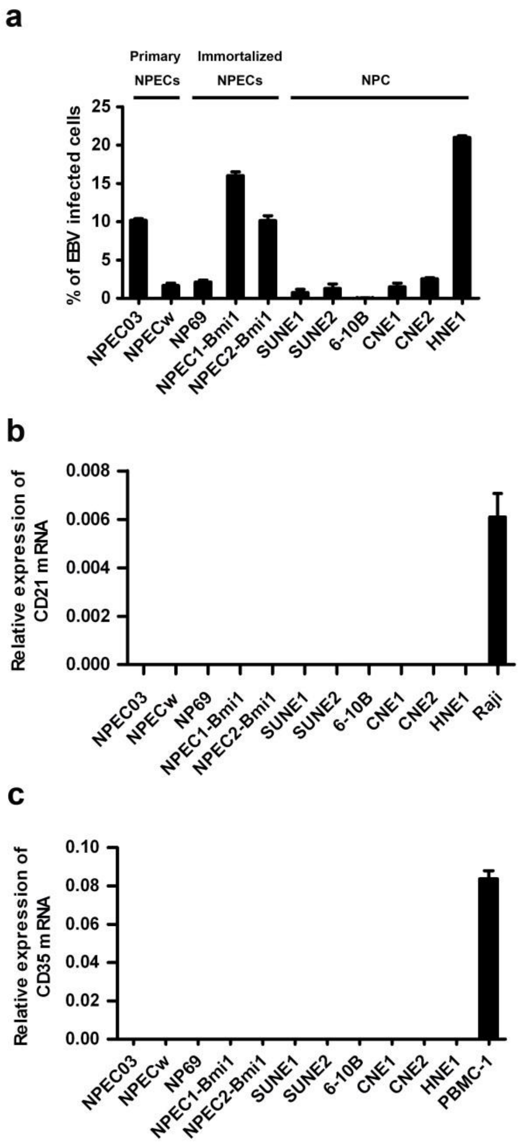

Supplementary Figure 3. Screening for nasopharyngeal epithelial cells relatively susceptible to EBV infection. (**a**) Primary nasopharyngeal epithelial cells (NPEC03 and NPECw at passage 5), immortalized nasopharyngeal epithelial cells (NP69, NPEC1-Bmi1 and NPEC2-Bmi1) and nasopharyngeal cancer cells (SUNE1, SUNE2, 6-10B, CNE1, CNE2 and HNE1) were incubated with cell-free EBV-GFP at a multiplicity of infection (MOI) of 2.5×10^3 virions per cell at 37°C for 2h. After brief wash with Hanks solution twice to remove unbound virus, cells were cultured in the fresh medium for 48h. The GFP-positive infected cells were determined by FACS. NPEC, nasopharyngeal epithelial cells; NPC,

nasopharyngeal cancer cells. n = 3. (**b,c**) CD21 and CD35 were negative in primary, immortalized nasopharyngeal epithelial cells and nasopharyngeal cancer cells. The mRNA levels of CD21 (**b**) and CD35 (**c**) were examined by real-time PCR. Raji (a human EBV-positive Burkitt's lymphoma cell line) was used as the positive control for CD21 expression. PBMC-1 (peripheral blood mononuclear cells) was used as the positive control for CD35 expression. n = 3. Values in all graphs are means ± s.e.m.

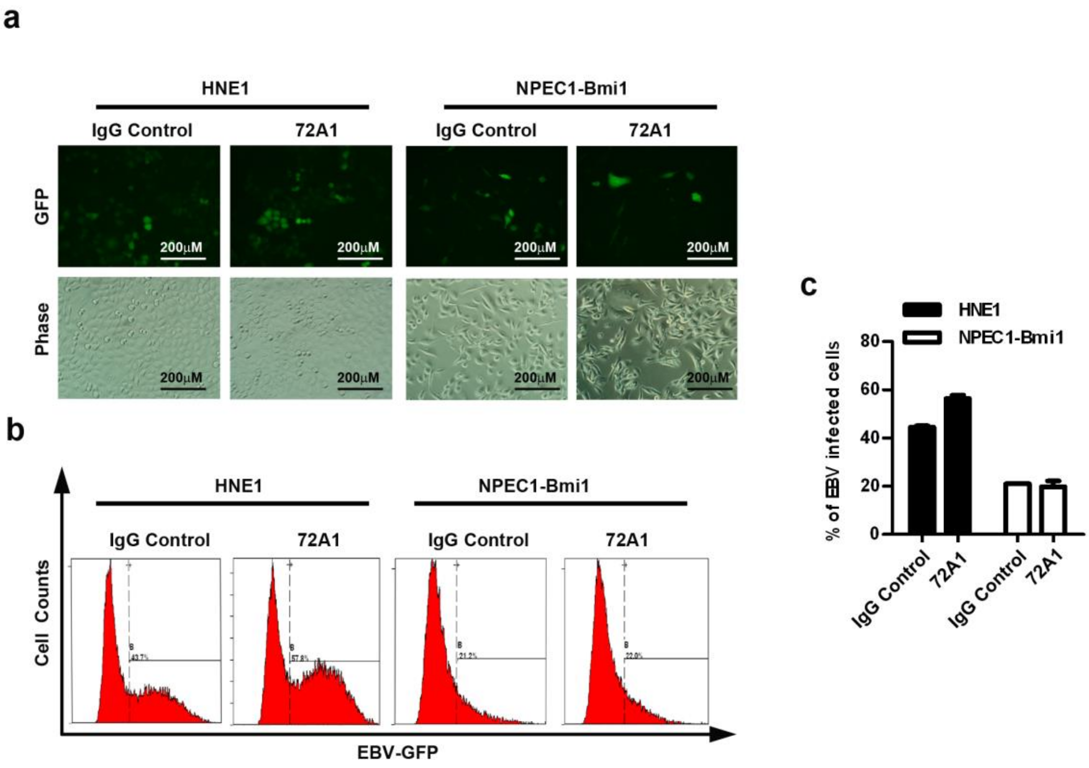

Supplementary Figure 4. Antibody against gp350 (72A1) has no effect on EBV infection of HNE1 and NPEC1-Bmi1 cells. EBV was preincubated with 25 μg ml^{-1} 72A1 mAb or mIgG for 1 h and subsequently infected of HNE1 and NPEC-Bmi1 cells for 2 h. After brief wash with Hanks solution twice to remove unbound virus, cells were cultured in the fresh medium for 48 h. The GFP-positive infected cells were determined by fluorescence and phase microscopy **(a**) or FACS **(b,c)**. The representative images **(a)**, histograms **(b)** and the percentage **(c)** of EBV infected cells were presented. Data represent three independent experiments. Values in all graphs are means ± s.e.m.

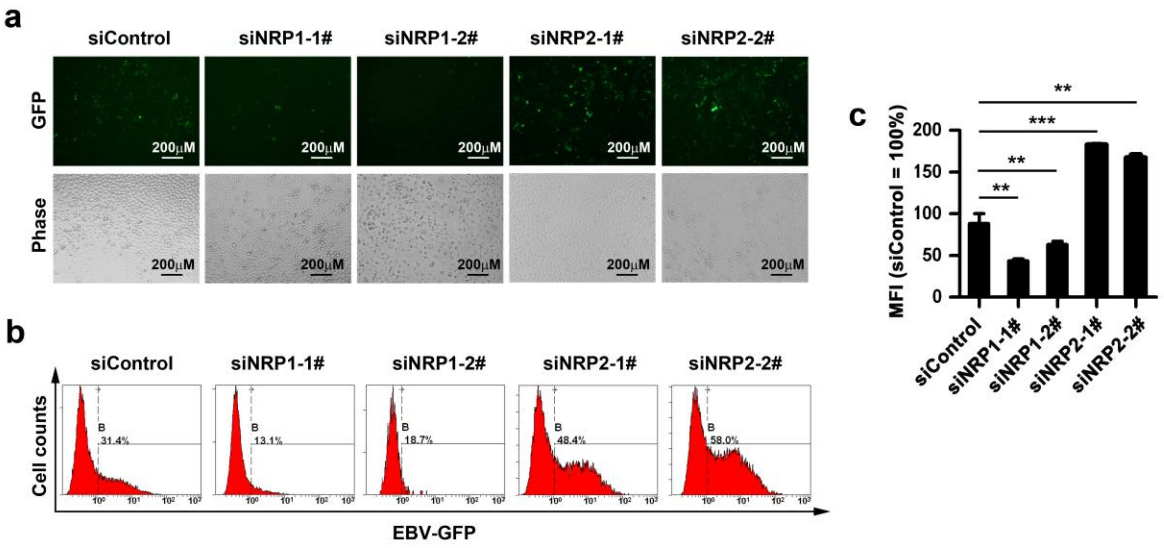

Supplementary Figure 5. Knockdown of NRP1 suppresses, whereas downregulation of NRP2 promotes EBV infection of HNE1 cells.

HNE1 cells transfected with siRNA against NRP1 or NRP2 for 48h were infected with EBV. Infection was analyzed by fluorescence and phase microscopy (**a**) or FACS (**b,c**) at 48 h post-infection. The representative images (**a**), histograms (**b**) and MFI (**c**) of EBV infected cells were presented, with MFI of siControl-transfected cells set to 100%. Data represent six independent experiments. Values in all graphs are means ± s.e.m. *** $P < 0.001$; ** $P < 0.01$; student's t-test.

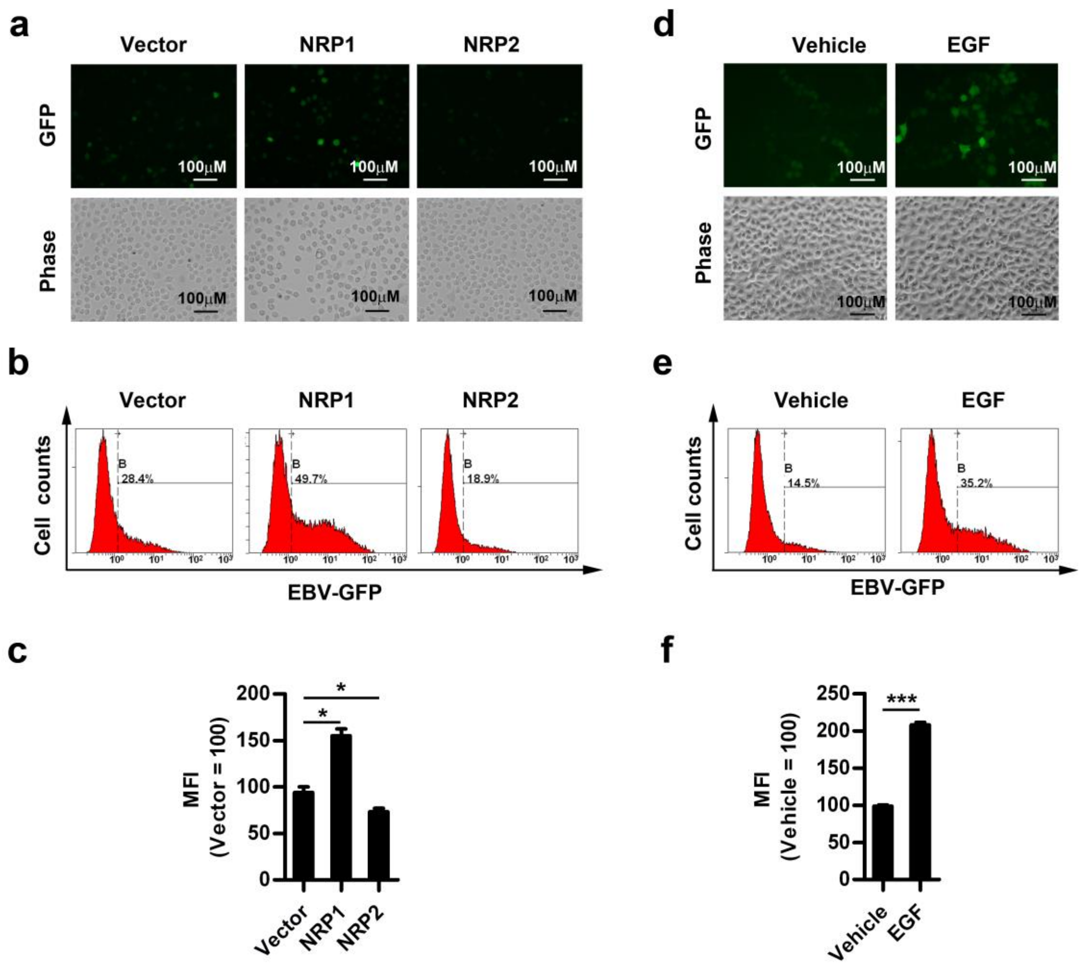

Supplementary Figure 6. NRP1 and EGF enhance EBV infection of HNE1 cells. HNE1 cells transfected with overexpression plasmids for NRP1 and NRP2 (**a**) or treated with EGF (**b**) were exposed to EBV for 2h. The GFP-positive infected cells were analyzed by fluorescence and phase microscopy (**a,d**) or FACS (**b,c,e,f**) at 24h post-infection. The MFI of EBV infected cells was presented (**c,f**), with the MFI of control cells set to 100. Data represent three independent experiments. Values in all graphs are means ± s.e.m. *** $P < 0.001$; * $P < 0.05$; student's t-test.

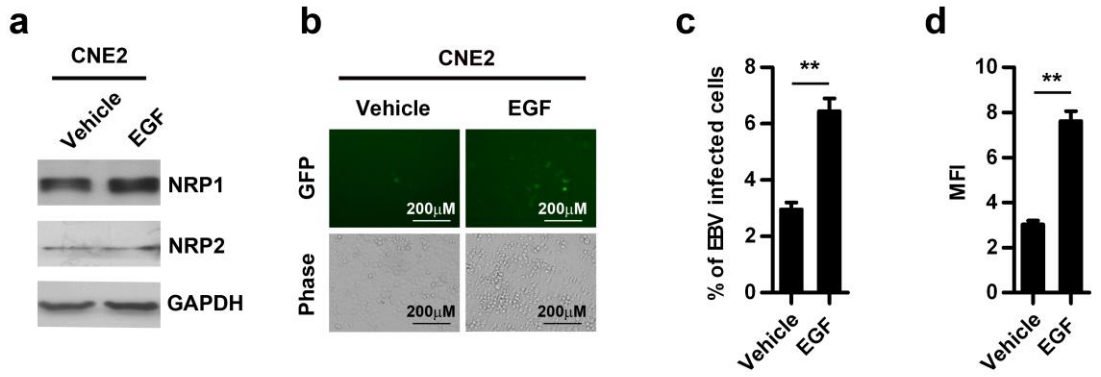

Supplementary Figure 7. EGF enhances EBV infection of CNE2 cells.

(**a**) EGF up-regulated NRP1 expression in CNE2 cells. CNE2 cells (5×10^4) were seeded in 24-well plate and followed by treatment with 10 ng ml^{-1} EGF for 24 h, and then subjected to western blotting assay for NRPs expression. GAPDH served as an internal control. (**b-d**) EGF enhanced EBV infection in CNE2 cells. EGF-treated CNE2 cells were infected with EBV. The GFP-positive infected cells were analyzed by fluorescence and phase microscopy (**b**) or FACS (**c,d**) at 48 h post-infection. The experiments were performed four times with similar results. Values in all graphs are means ± s.e.m. ** $P < 0.01$; student's t-test.

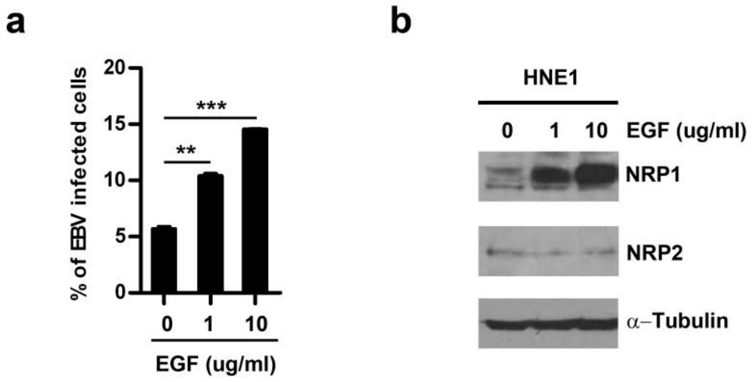

Supplementary Figure 8. EGF promotes EBV infection and the expression of NRP1 in a dose-dependent manner. (**a**) EGF enhanced EBV infection in HNE1 cells in a dose-dependent manner. HNE1 cells were incubated with the indicated concentrations of EGF and were infected with EBV. The GFP-positive infected cells were analyzed by FACS at 48 h post-infection. (**b**) EGF up-regulated NRP1 expression in HNE1 cells in a dose-dependent manner. HNE1 cells (5×10^4) were seeded in 24-well plate and followed by treatment with the indicated concentration of EGF for 24 h, and then subjected to western blotting assay for NRP1 and NRP2 expression. α-Tubulin served as an internal control. The experiments were performed three times with similar results. Values in all graphs are means ± s.e.m. *** P < 0.001; ** P < 0.01; student's t-test.

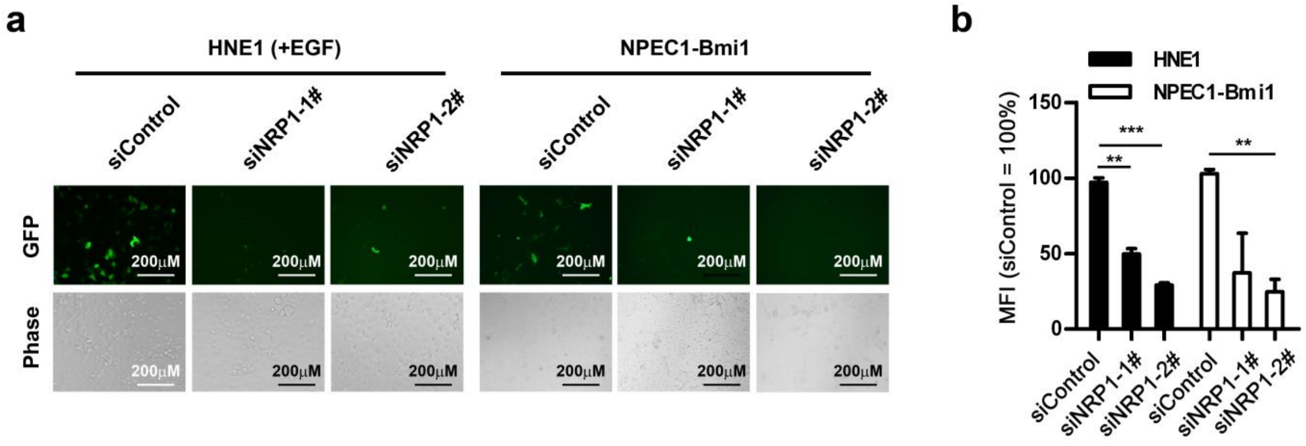

Supplementary Figure 9. Knockdown of NRP1 inhibits EBV infection of EGF-treated HNE1 and NPEC1-Bmi1 cells. EGF-treated HNE1 and NPEC1-Bmi1 cells maintained in KSF medium supplemented with EGF were transfected with siRNA against NRP1 for 48 h, and then infected with EBV. EBV infection was analyzed by fluorescence and phase microscopy **(a)** and FACS **(b)** at 48 h post-infection. The MFI of siControl-transfected cells were set to 100%. Data represent three independent experiments. Values in all graphs are means ± s.e.m. *** $P < 0.001$; ** $P < 0.01$; student’s t-test.

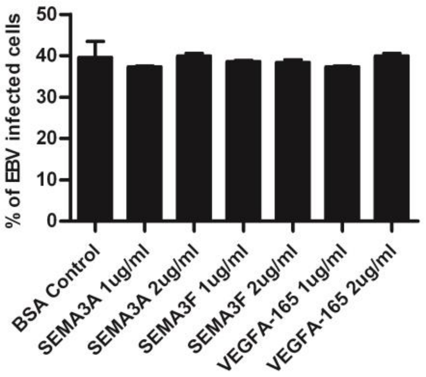

Supplementary Figure 10. The ligands for NRP1 have no effect on EBV infection. HNE1 cells (2.5×10^4) were seeded in 48-well plate for 16 h and were incubated with the indicated doses of the NRP1 ligands (SEMA3A, SEMA3F or VEGFA) for 1 h, and then infected with EBV. EBV infection was analyzed by FACS at 48 h post-infection. Values in all graphs are means ± s.e.m. n = 3.

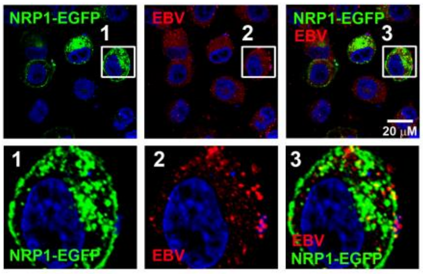

Supplementary Figure 11. EBV co-localized with NRP1. HNE1 cells transiently transfected with the expression plasmids for NRP1 (NRP1-EGFP) for 24 h and were then infected with cell-free EBV. Expressions of NRP1-EGFP were visualized as green. EBV was stained with mouse antibody against gp350, followed by a secondary antibody conjugated with Alexa Fluor-594 (red). Nuclei were stained with DAPI (blue). The images of the boxed areas labeled as 1, 2 and 3 were enlarged and shown in the bottom panels. The representative immunofluorescence images were presented.

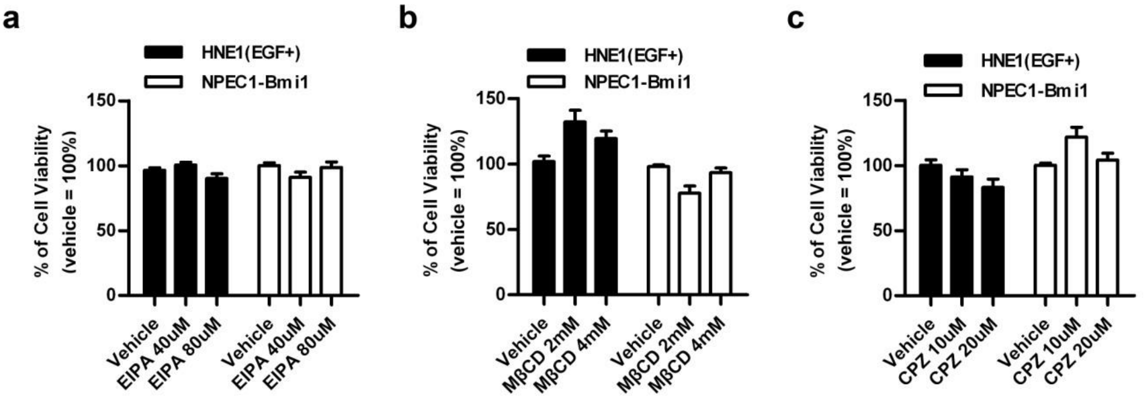

Supplementary Figure 12. The endocytic inhibitors have no obvious effect on cell viability. (**a-c**) EGF-treated HNE1 cells and NPEC1-Bmi1 cells were incubated with the indicated doses of EIPA (**a**), MβCD (**b**) and CPZ (**c**) for 150 min, the cells were then washed with Hanks solution twice and cultured for 48 h until MTT analysis for the cell viability. Data represent three times independent experiments. Graphs show mean ± s.e.m.

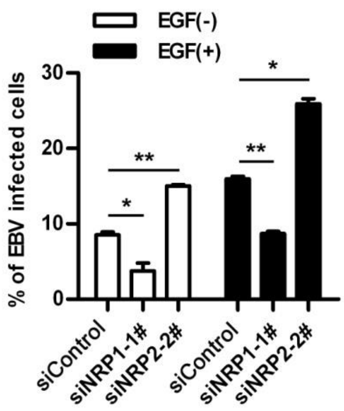

Supplementary Figure 13. Facilitating role of NRP1 and EGF in the cell-to-cell contact mediated EBV infection. HNE1 cells transfected with the indicated siRNA duplexes were cultured in the absence (open bar) or presence (solid bar) of EGF for 48 h. After replacement with fresh medium, HNE1 cells were cocultured with goat anti-human IgG-induced Akata cells carrying EBV for 36 h. The percentage of EBV infected HNE1 cells was analyzed by FACS. Data represent three independent experiments. Values in all graphs are means ± s.e.m. ** $P < 0.01$; * $P < 0.05$; student's t-test.

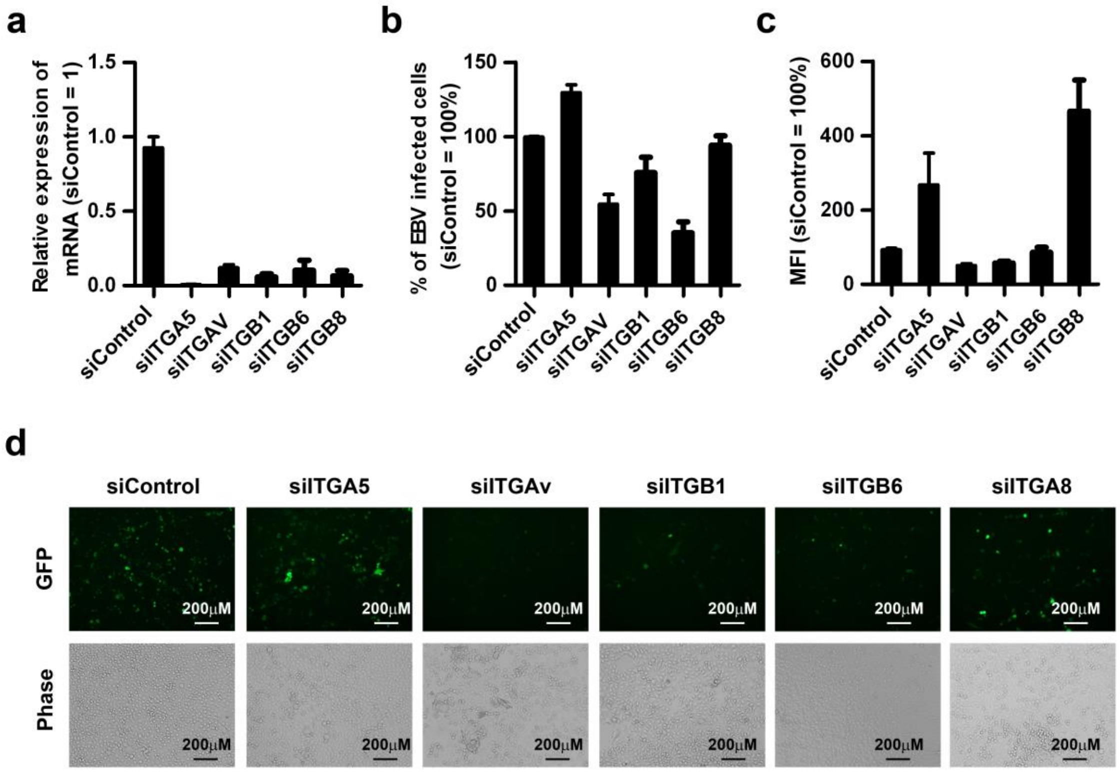

Supplementary Figure 14. Knockdown of ITGAV, ITGB1 and ITGB6 inhibit EBV infection in HNE1 cells.

(**a**) Indicated integrins were silenced in HNE1 cells. HNE1 cells were transfected with siRNA duplexes targeting α5, β1, αv, β6 and β8 for 48 h, followed by real-time PCR analyses for their respective target genes. GAPDH was used as an internal standard. (**b-d**) Transfected HNE1 cells were infected with EBV at a MOI of 5×10^3. The GFP-positive infected cells were analyzed by FACS (**b,c**) and microscopy (**d**) at 48 h post-infection. siControl-transfected cells were set to 100%. The percentage (**b**) or MFI (**c**) of EBV infected cells were presented. Data represent three independent experiments. Values in all graphs are means ± s.e.m.

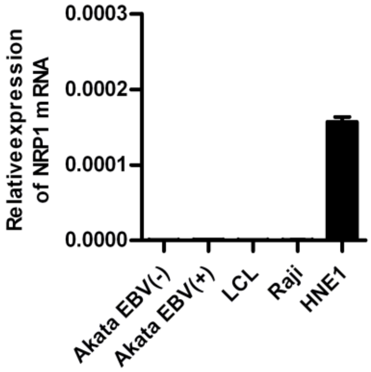

Supplementary Figure 15. NRP1 is not expressed in the examined B cell strains. The mRNA levels of NRP1 in B cell strains (Akata, LCL and Raji) were determined by real-time PCR. Akata EBV (-), EBV negative Akata cells; Akata EBV (+), EBV positive Akata cells. GAPDH served as an internal control, n = 3, Values in all graphs are means ± s.e.m.

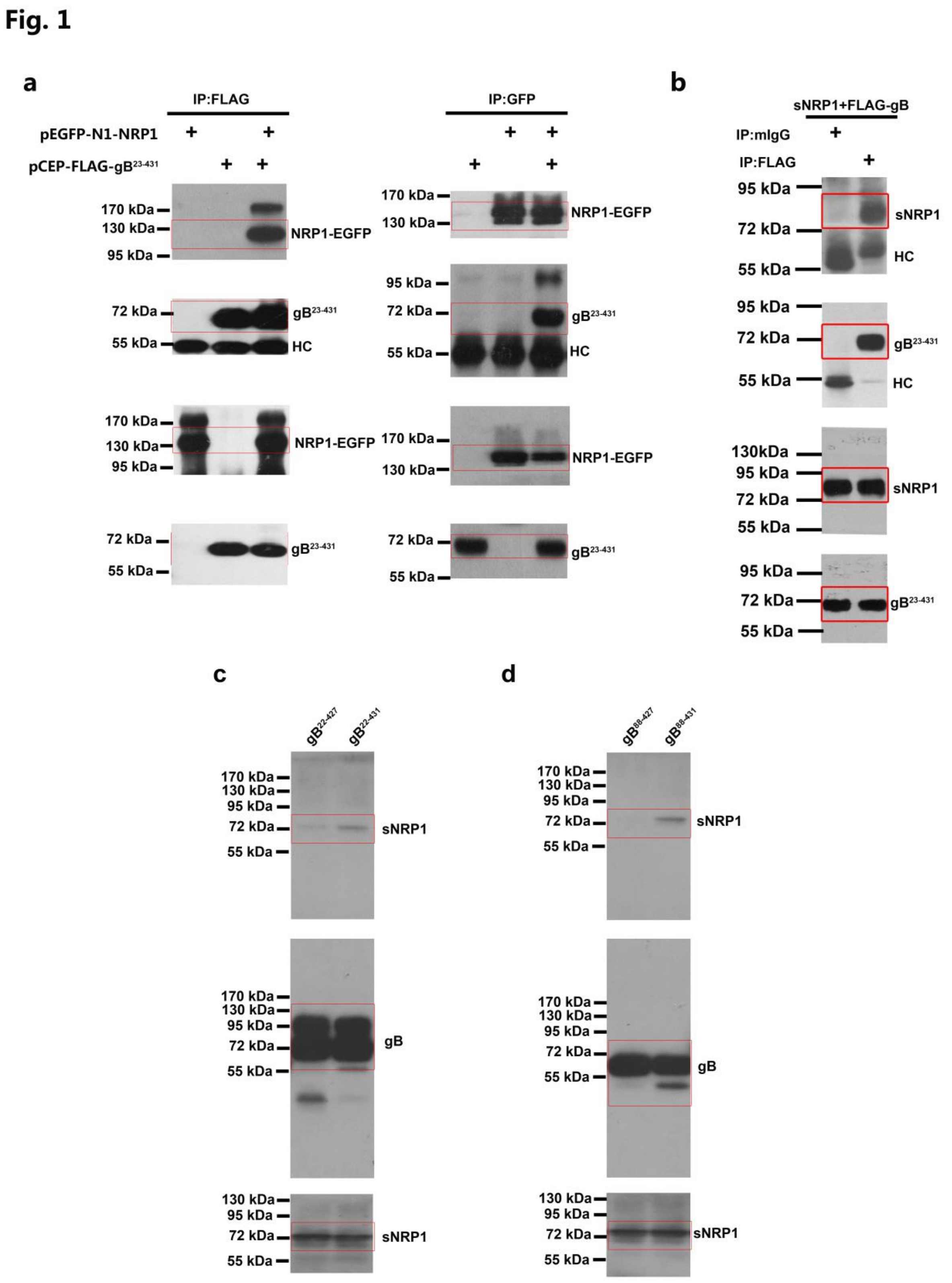

Supplementary Figure 16. Full scans for Figure 1.

Fig. 2

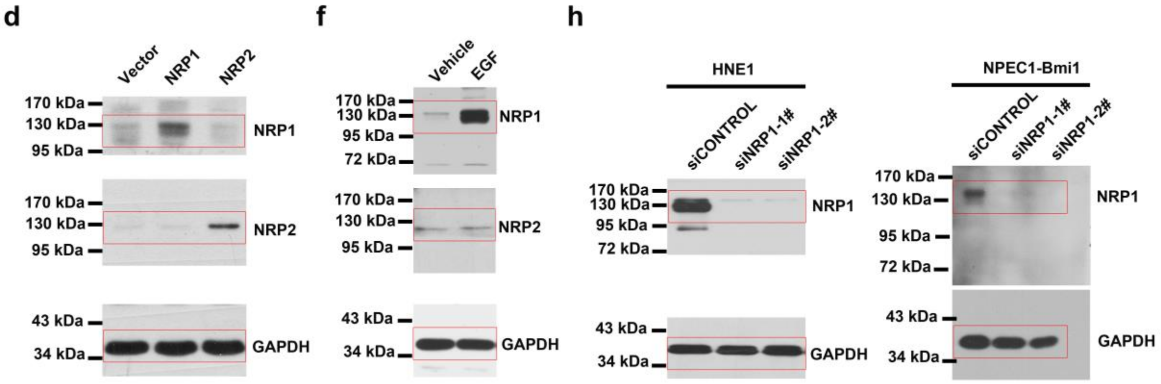

Fig. 3

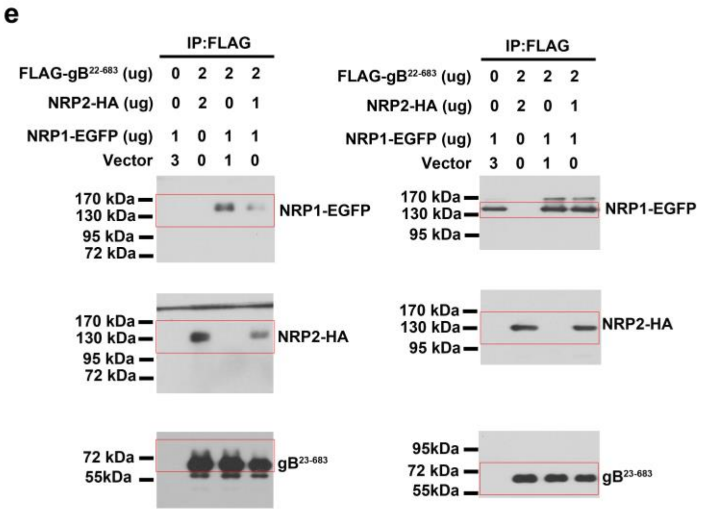

Supplementary Figure 17. Full scans for Figure 2 and 3.

Fig. 6

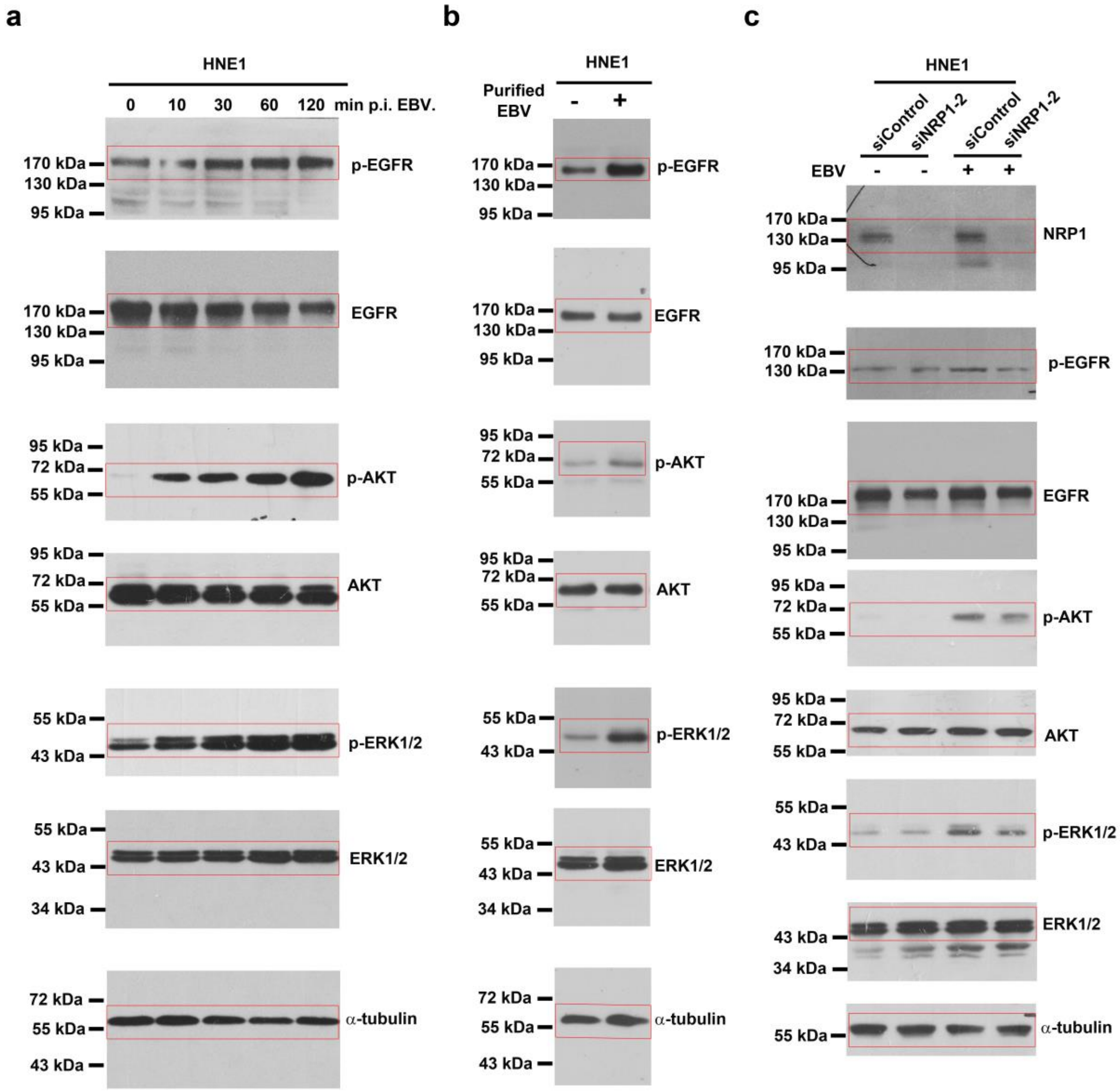

Supplementary Figure 18. Full scans for Figure 6.

Supplementary Fig.1

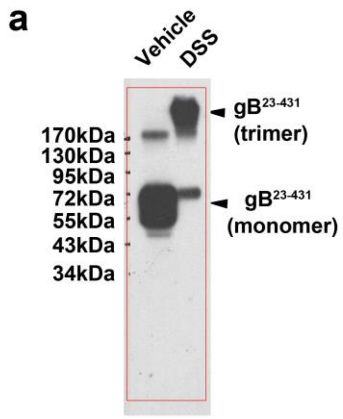

Supplementary Fig. 2

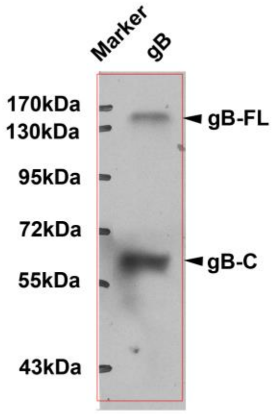

Supplementary Fig. 7

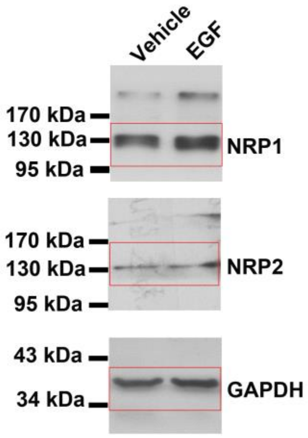

Supplementary Fig. 8

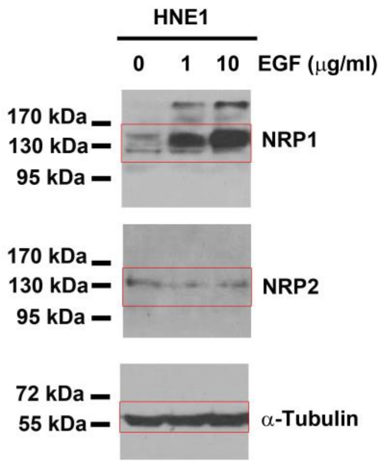

Supplementary Figure 19. Full scans for Supplementary Figure 1, 2, 7 and 8.

Supplementary Table 1. Sequences of primers for cloning

Name	Sense<br>Antisense	Enzyme<br>Site	Primers (5' - 3')
pEGFP-N1-NRP1	Sense	*Eco*RI	CGGAATTCGAGAATGGAGAGGGGGCTGC
	Antisense	*Bam*HI	CGGGATCCAGTGCCTCCGAATAAGTACTCTGTG
pMSCV-NRP1	Sense	*Xho*I	CCGCTCGAGGAGAATGGAGAGGGGGCTGC
	Antisense	*Eco*RI	CGGAATTC CTGTCTGCCTTCATGCCTCC
pMSCV-NRP1-Mcherry	Sense	*Xho*I	CCGCTCGAGGAGAATGGAGAGGGGGCTGC
	Antisense	*Eco*RI	CGGAATTCTGCCTCCGAATAAGTACTCTGTG
pET32a-FLAG-NRP1^{ABC}	Sense	*Bam*HI	CGGGATCCATGGACTACAAAGACGATGACGACAAGCTT CGCAACGATAAATGTGGCGATAC
	Antisense	*Xho*I	CCGCTCGAG TATGATGGTGATGAGGATGGGGTC
pEGFP-N1-NRP2	Sense	*Hind* III	CCCAAGCTT ATGGATATGTTTCCTCTCACCTGG
	Antisense	*Kpn* I	GGGGTACCAATGCCTCGGAGCAGCACTTTTG
pLNCX2-NRP2	Sense	*Hind* III	CCCAAGCTTATGGATATGTTTCCTCTCACCTGG
	Antisense	*Sal* I	ACGCGTCGACTCATGCCTCGGAGCAGCACTT
pLenti-NRP2	Sense	*Kpn* I	GGGGTACCATGATGGATATGTTTCCTCTCACCTGG
	Antisense	*Xba* I	GCTCTAGATGCCTCGGAGCAGCACTTTTG
pCEP-his-FLAG-gB$^{23-683}$	Sense	*Nhe* I	CTAGCTAGCGACTACAAAGACGATGACGACAAGCTTGCGCAGACCCCAGAGCAGCC
	Antisense	*Bam*HI	CGGGATCC TTATCACAGATCCTCTTCTGAGATGAG
pCEP-his-FLAG-gB$^{23-431}$	Sense	*Nhe*I	CTAGCTAGCGACTACAAAGACGATGACGACAAGCTTGCGCAGACCCCAGAGCAGCC
	Antisense	*Bam*HI	CGGGATCCTTATCACCTCCGGCGCCTCAGAACGGC
pCEP-his-FLAG-gB$^{23-427}$	Sense	*Nhe*I	CTAGCTAGCGACTACAAAGACGATGACGACAAGCTTGCGCAGACCCCAGAGCAGCC
	Antisense	*Bam*HI	CGGGATCC TTATCACAGAACGGCGGCGGAGGTGCTC
pCEP-his-FLAG-gB$^{89-431}$	Sense	*Nhe*I	CTAGCTAGCGACTACAAAGACGATGACGACAAGCTTATTATTCCCTACTCGTTTAAGG
	Antisense	*Bam*HI	CGGGATCCTTATCACCTCCGGCGCCTCAGAACGGC
pCEP-his-FLAG-gB$^{89-427}$	Sense	*Nhe*I	CTAGCTAGCGACTACAAAGACGATGACGACAAGCTTATTATTCCCTACTCGTTTAAGG
	Antisense	*Bam*HI	CGGGATCC TTATCACAGAACGGCGGCGGAGGTGCTC
pEGFP-C2-SNX5	Sense	*Eco*RI	GGAATTCATGGCCGCGGTTCCCGAGTTGC
	Antisense	*Bam*HI	CGGGATCCTTATTCTTGAACAAGTCAATACAGC
pcDNA3.1(+)-NRP1$^{30-636}$	Sense	*Bam*HI	CGGGATCC ATGATGCGCAACGATAAATGTGGCGATAC
	Antisense	*Eco*RI	GGAATTCTCAAGCGTAGTCTGGGTCGTATGGGTAGTCTATGACCGTGGGCTTTTCTGTG
pGEX-6p-1-NRP1^{ABC}	Sense	*Bam*HI	CGGGATCCATGGACTACAAAGACGATGACGACAAGCTTCGCAACGATAAATGTGGCGATAC
	Antisense	*Xho*I	CCGCTCGAG TATGATGGTGATGAGGATGGGGTC
pGEX-6p-1-NRP2^{ABC}	Sense	*Sal*I	ACGCGTCGACGGAGGTCGTTTGAATTCCAAAGATG
	Antisense	*Sal*I	ACGCGTCGACTCAGCTCTTTTCTTTGTCGGTCGAG

Supplementary Table 2 . Sequence of the oligonucleotides for siRNA

Name	GenBank Accession No.	Cat. No.	Sequence (5' - 3')
siControl		D-001220-01-20	
siITGA5	NM_002205.2	M-008003-02	ACACGUUGCUGACUCCAUU
			GAACGAGUCAGAAUUUCGA
			CAAACGCUCCCUCCCAUAU
			UGAAGAUGCCCUACCGAAU
siITGAv	NM_002210.3	M-004565-03	GGAUGAAUCUGAAUUUAGA
			CCAUGUAGAUCACAAGAUA
			CGACAAAGCUGAAUGGAUU
			CCGAAACAAUGAAGCCUUA
siITGB1	NM_002211.3;	M-004506-00	GAACAGAUCUGAUGAAUGA
			CAAGAGAGCUGAAGACUAU
			GAAGGGAGUUUGCUAAAUU
			CCACAGACAUUUACAUUAA
siITGB6	NM_000888.3	M-008012-01	GCUAAAGGAUGUCAAUUAA
			GAACGGCUCUUUCCAGUGU
			CAUCUCAGCUUAUGAAGAA
			GCCAACCCUUGCAGUAGUA
siITGB8	NM_002214.2	M-008014-02	CUGCAAACCUCAAUAAUUU
			GCAGAAACGUGACGAGCAA
			UGGAAACGAUUUAUCUAGA
			GAUCAGACGUCUCAUCUCG
siNRP1-1#	NM_003873.5		AACACCTAGTGGAGTGATAAA
siNRP1-2#			AACAGCCTTGAATGCACTTAT
siNRP2-1#	NM_003872.2		CCCAACCAGAAGATTGTCC
siNRP2-2#			AGAACTGCGAGTGGATTGT
siEGFR	NM_005228.3	siB06112493553	GGCTGGTTATGTCCTCATT
siMET	NM_000245.2	siG11214164750	CTACTTATGTGAACGTAAA

Supplementary Table 3 Primers for real-time PCR

Name	GenBank Accession No.	Sense Primers (5' - 3')	Antisense Primers (5' - 3')
NRP1	NM_003873.5	CAGAAAAGCCCACGGTCAT	CAGCCAAATTCACAGTTAAAACC
NRP2	NM_003872.2	AAGTCTCCTACAGCCTAAACGG	GATGTCAGGGGTGTCATAGTGC
CD21	NM_001006658	TCGCGTGATTAGGTGTCATACTG	TCCCGTTAGGGGTCTTAGGC
CD35	NM_000573.3	GCGGTTGTGGTGCTGCTT	CCAGGGCGGCATTCATAG
ITGA5	NM_002205.2	AGGCTTCAGTGCCGAGTTCA	GGTGACATAGCCGTAAGTGAGGTT
ITGAV	NM_002210.3	GAGCAATTCGACGAGCACTGT	GATTCATCCCGCAGATACGCT
ITGB1	NM_002211.3	GTGGTTGCTGGAATTGTTCTTATT	TTTTCCCTCATACTTCGGATTGAC
ITGB6	NM_000888.3	AACCTGTATCCCCTTTCGTGA	TGGCAAAATGTGCTTGAATCC
ITGB8	NM_002214.2	CATAGTGGTGCCCAATGACGG	CAGCCTTTGATTCTATTTCACCAG
EGFR	NM_005228.3	AGGCACGAGTAACAAGCTCAC	ATGAGGACATAACCAGCCACC
MET	NM_000245.2	GTAAGTGCCCGAAGTGTAAGC	GTTCTGAGATGAATTAGGAAACTGAT
GAPDH	NM_002046.4	CTCCTCCTGTTCGACAGTCAGC	CCCAATACGACCAAATCCGTT
EBV-BamHI-W	AY961628.3	CCCAACACTCCACCACACC	TCTTAGGAGCTGTCCGAGGG
GAPDH DNA	NG_007073.2	CCCCACACACATGCACTTACC	CCTAGTCCCAGGGCTTTGATT
EBV-BamHI-W-FAM-probe		FAM-CACACACTACACACACCCACCCGTCTCTAMRA	
GAPDH DNA-FAM-probe		FAM-AAAGAGCTAGGAAGGACAGGCAACTTGGCBHQ1	
